# Luminal Progenitors Restrict Their Lineage Potential during Mammary Gland Development

**DOI:** 10.1371/journal.pbio.1002069

**Published:** 2015-02-17

**Authors:** Veronica Rodilla, Alessandro Dasti, Mathilde Huyghe, Daniel Lafkas, Cécile Laurent, Fabien Reyal, Silvia Fre

**Affiliations:** 1 Department of Genetics and Developmental Biology, Institut Curie, Paris, France; 2 Centre National de la Recherche Scientifique CNRS UMR3215, Paris, France; 3 Institut National de la Santé et de la Recherche Médicale Inserm U934, Paris, France; 4 Department of Translational Research, Institut Curie, Paris, France; 5 Department of Surgery, Institut Curie, Paris, France; B.C. Cancer Agency, CANADA

## Abstract

The hierarchical relationships between stem cells and progenitors that guide mammary gland morphogenesis are still poorly defined. While multipotent basal stem cells have been found within the myoepithelial compartment, the in vivo lineage potential of luminal progenitors is unclear. Here we used the expression of the Notch1 receptor, previously implicated in mammary gland development and tumorigenesis, to elucidate the hierarchical organization of mammary stem/progenitor cells by lineage tracing. We found that Notch1 expression identifies multipotent stem cells in the embryonic mammary bud, which progressively restrict their lineage potential during mammary ductal morphogenesis to exclusively generate an ERα^neg^ luminal lineage postnatally. Importantly, our results show that Notch1-labelled cells represent the alveolar progenitors that expand during pregnancy and survive multiple successive involutions. This study reveals that postnatal luminal epithelial cells derive from distinct self-sustained lineages that may represent the cells of origin of different breast cancer subtypes.

## Abstract 2

Stem cells in the embryonic mammary gland that express the Notch1 receptor are initially multipotent and highly regenerative, but they progressively restrict their lineage potential to the lumen of the mammary duct, where they may give rise to breast cancer.

## Author Summary

Tissue-specific stem cells are believed to be multipotent, thus able to generate all cell types of their tissue of origin. In the mammary gland epithelium, however, the existence of multipotent versus unipotent adult stem cells is currently under debate. In this study, we have identified and characterized a population of mammary luminal progenitors that express the Notch1 receptor. Using lineage tracing experiments, we found that these cells are self-sustained unipotent adult progenitors with high self-renewal capacity. Although they lack estrogen and progesterone hormone receptors, these cells are highly responsive to hormones. Importantly, Notch1-expressing cells are multipotent during embryonic mammary development, when they can give rise to all mammary cell types, while they become lineage-restricted postnatally. The cells characterized in this study also present extensive plasticity, as they can repopulate the entire mammary gland in transplantation experiments. Our study reveals that the Notch1 receptor is a specific marker for the identification of luminal progenitors that lack expression of hormone receptors and that can be critical for breast cancer initiation.

## Introduction

The mammary gland is composed of two epithelial compartments: an inner layer, which contains luminal cells, and an outer myoepithelial layer in direct contact with the basal membrane. These two cell lineages originate from mammary stem cells (MaSC) that were initially found to reside in the basal compartment, as only these cells have been reported to efficiently regenerate a complete mammary gland in transplantation assays [1,2]. However, more recent in vivo lineage tracing studies using promoters of specific cytokeratins expressed either in the luminal or in the myoepithelial compartment have led to conflicting results regarding the plasticity of multipotent mammary stem cells in the adult mammary gland. While Visvader and colleagues showed that some basal cells are able to generate both luminal and myoepithelial cells in the adult gland [3], Blanpain and colleagues indicated that after birth, the two lineages originate from distinct unipotent progenitors [4].

Luminal cells have been roughly subdivided into ductal and alveolar subtypes, and they have received increasing attention in the past few years, partly because they are thought to be the cells of origin for the vast majority of breast tumors [5,6]. Although several surface markers for luminal progenitors have been reported, such as the Stem cell antigen-1 (Sca1), Prominin1 (CD133) [7], α2-integrin (CD49b) [8], E74-like factor 5 (Elf5) [9,10], CD61 [11], CD14 [8], c-Kit [12], Notch2 [13], and Notch3 [14], their functional significance and the hierarchical relationships between different subsets of luminal cells are still unclear. Some luminal progenitors are responsible for forming alveolar buds during pregnancy, when milk-secreting cells are specified [15]. The process of alveologenesis is orchestrated by consecutive waves of hormones that drive extensive proliferation and remodeling of the mammary epithelium at pregnancy. Luminal cells expressing the ovarian steroid hormone receptors Estrogen Receptor-α (ERα) and Progesterone Receptor (PR), designated hormone-sensing cells, are directly stimulated by circulating hormones and induce proliferation of neighboring cells, referred to as hormone-responsive cells, that lack expression of ERα or PR [16,17]. While luminal progenitors are believed to be largely ERα^neg^, a small fraction of ERα^pos^ cells have also been found to have progenitor features, at least in vitro [7,18].

With the aim of unraveling the cellular heterogeneity of the luminal epithelium, we have used the Notch1 promoter to genetically label and follow the fate of Notch1-expressing cells during mammary gland development and adult homeostasis in vivo. Indeed, the Notch signaling pathway is involved in stem cell maintenance, preservation of progenitor pools, and control of cell differentiation in a variety of tissues [19,20]. In the mammary gland, the Notch1 receptor has been found expressed in the luminal compartment, and its ectopic activation in basal cells induces luminal cell commitment [21]. Nevertheless, it is still unknown which types of luminal cells physiologically express this Notch paralogue.

During embryonic development, we show by lineage tracing that Notch1-expressing cells are multipotent and can produce all mammary lineages, including myoepithelial and both ERα^pos^ and ERα^neg^ luminal cells. The potency of these progenitors is then restricted perinatally, resulting in unipotent cells that exclusively maintain their own ERα^neg^ lineage. Indeed, we find that in the postnatal mammary gland, Notch1 marks cells that invariably lack expression of the ERα and PR hormone receptors, displaying high self-renewal capacity throughout life. These cells are rapidly mobilized in response to hormones and are crucial for alveolar formation at pregnancy. Importantly, the high regeneration capacity of these cells is revealed in transplantation assays, demonstrating that Notch1-expressing cells can generate complete mammary outgrowths consisting of both myoepithelial and luminal cells. Our results collectively suggest that, differently from embryonic mammary stem cells, in the pubertal and adult mammary gland, ERα^neg^ and ERα^pos^ luminal cells represent independent and self-sustained lineages, derived from distinct unipotent progenitors.

## Results

### Notch1 Marks Multipotent Embryonic Progenitors in the Mammary Bud

**Fig 1:**
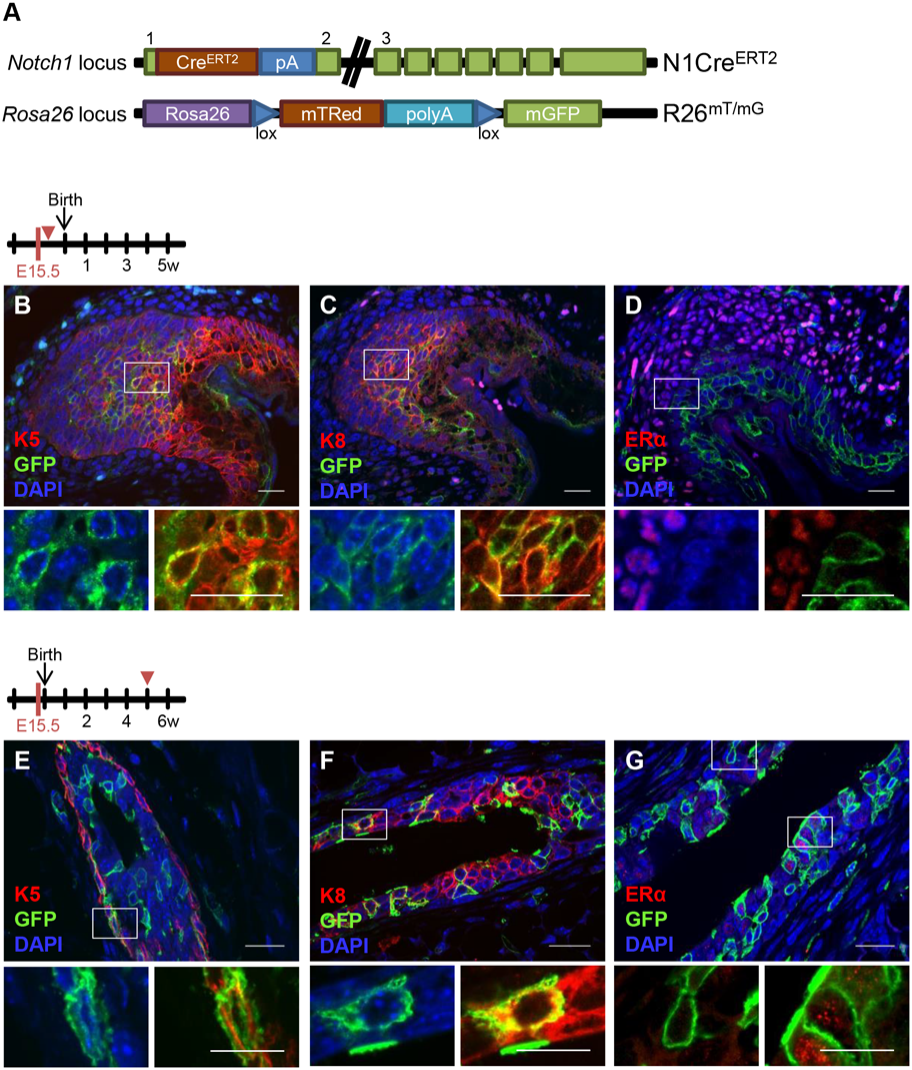
Notch1 is expressed in multipotent embryonic stem cells. **(A)** Schematic representation of the Notch1-CreERT2^SAT^ knock-in mice (referred to as N1Cre^ERT2^) and Rosa26^mTomato/mGFP^ reporter mice (called R26^mTmG^) used in this study. Pregnant females were induced with tamoxifen to label their embryos at embryonic day E15.5 and double transgenic N1Cre^ERT2^R26^mTmG^ littermates were analyzed 24 h later **(B–D)** or 5 wk after birth (**E–G**). **(B–D)** Representative embryonic mammary bud sections show that Notch1^pos^ cells (marked by GFP in green) express both myoepithelial (K5, in red in B) and luminal markers (K8, in red in C) and they are negative for ERα (in red in D), *n* = 2. (**E–G**) Representative pubertal mammary gland sections show that Notch1-derived clones (in green) contain myoepithelial (K5^pos^ in red in E) and luminal cells (K8^pos^ in red in F) as well as ERα^pos^ and ERα^neg^ cells (ERα in red in G), *n* = 3. 4',6-diamidino-2-phenylindole (DAPI) stains DNA in blue. Scale bars correspond to 20 µm in B–G, and 10 µm in the magnifications of the insets.

Previous work has shown that K14^pos^ basal mammary cells can give rise to all mammary cell types during embryonic development, but they restrict their potential and do not contribute to the luminal lineage in the postnatal mammary gland [4]. As Notch signaling has been shown to dictate cell fate decisions in the mouse mammary gland [21], we analyzed the differentiation potential of Notch1-expressing cells during embryonic development, when the embryonic mammary placodes invaginate to form mammary buds [22]. To this end, we used Notch1-CreERT2^SAT^ mice [23] crossed to a double fluorescent reporter line, Rosa26^mTmG^ [24], referred to as N1Cre^ERT2^R26^mTmG^ (Fig. 1A), which we previously characterized in the mouse intestine [23], to genetically label with membrane-bound Green Fluorescent Protein (GFP) Notch1-expressing cells and their progeny in vivo in the mammary gland. The CreERT2 inducible system permits the identification of Notch1-expressing cells when mice receive a single dose of tamoxifen and are analyzed 24 h later (pulse), but it also allows following the clonal progeny derived from initially labeled cells, by analyzing mice at longer time points after tamoxifen administration (chases) (S1 Fig.). To induce the activity of the CreERT2 recombinase, we used 0.1 mg of tamoxifen/g of mouse body weight, a dose that does not delay or alter mammary gland development [3].

When we administered a single dose of tamoxifen to pregnant females 15.5 d post-coitum (dpc), and analyzed the double transgenic N1Cre^ERT2^R26^mTmG^ embryos 24 h later, we found that GFP-marked cells co-expressed both myoepithelial cytokeratin 5 (K5) and luminal cytokeratin 8 (K8) markers (Fig. 1B,C and S2A Fig.), confirming that the two epithelial lineages are not yet resolved at this developmental stage [25,26]. The analysis of the cell lineages derived from these embryonic progenitors in N1Cre^ERT2^R26^mTmG^ mice 5 wk after birth revealed that the GFP-labeled progeny contained both myoepithelial cells (marked by the expression of K5, K14 and p63) (Fig. 1E and S2B,C Fig.) and K8^pos^ luminal cells (Fig. 1F). Corroborating these results, flow cytometry analysis of these cells, using the established CD24 and CD29 surface markers to resolve the luminal (CD24^+^CD29^low^) and myoepithelial (CD24^+^CD29^high^) cell populations, confirmed that the GFP-labeled progeny was composed by both cell types (S2E Fig.). In addition, while embryonic mammary cells lack expression of ERα (Fig. 1D) [27], GFP-marked clones were composed of both ERα^pos^ and ERα^neg^ cells (Fig. 1G) and both PR^pos^ and PR^neg^ cells (S2D Fig.). The presence of GFP-marked progeny containing all mammary lineages implies that the Notch1 receptor is expressed in a pool of embryonic mammary stem cells, co-expressing basal and luminal markers (S2A Fig.), which is able to give rise to all mammary lineages after birth.

### Notch1 Expression Is Restricted To ERα^neg^ and PR^neg^ Luminal Cells in the Postnatal Mammary Gland

**Fig 2:**
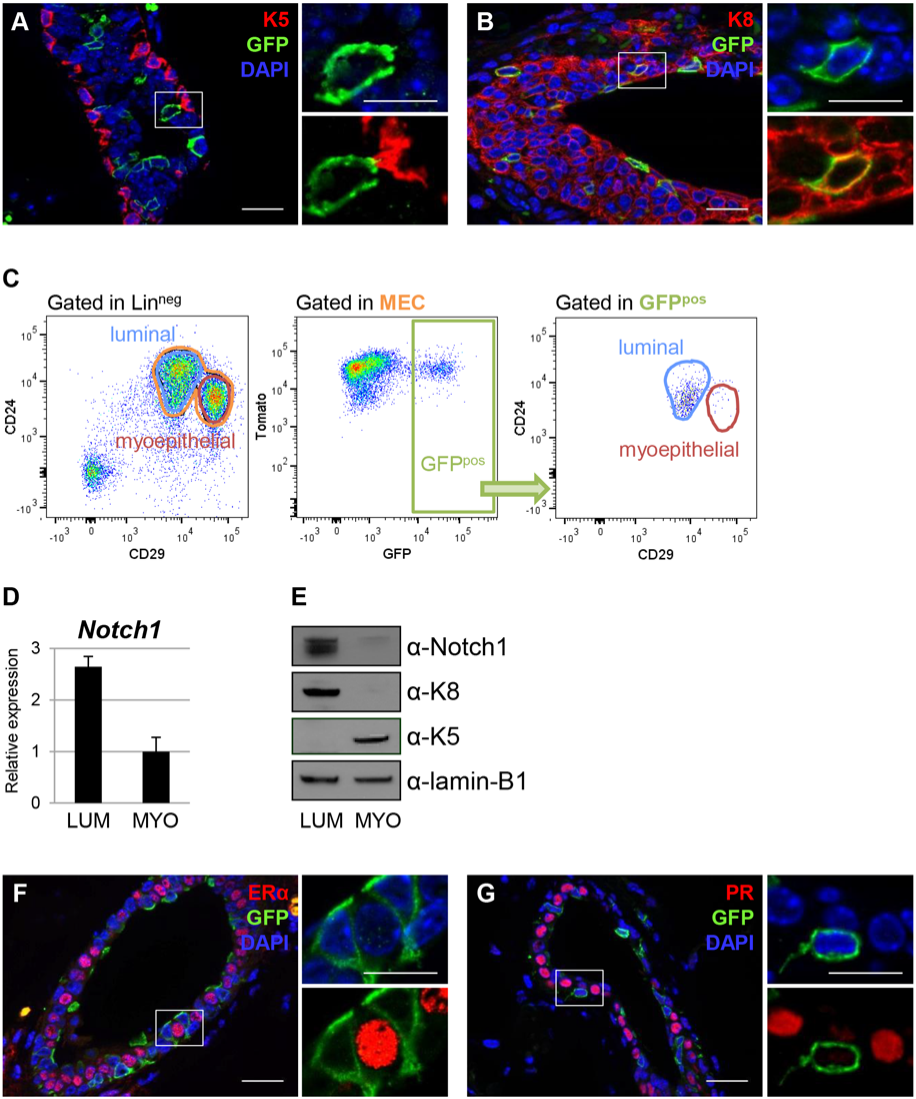
Notch1 expression in the postnatal mammary gland is restricted to ERα^neg^ and PR^neg^ luminal cells. **(A–B)** Representative sections of ducts from pubertal (6-wk-old) N1Cre^ERT2^R26^mTmG^ females analyzed 24 h upon tamoxifen injection. Immunofluorescence was performed with anti-K5 antibodies (in red in A), anti-K8 (in red in B), anti-GFP (to reveal Notch1-marked cells in green) and DAPI stains DNA in blue; *n* = 3. **(C)** FACS plots of 6-wk-old N1Cre^ERT2^R26^mTmG^ females analyzed 24 h upon tamoxifen injection. Dissociated mammary cells were gated as Lin^neg^ cells (CD45/CD31/Ter119)^neg^, and then as mammary epithelial cells (MEC) using the CD24 and CD29 markers, allowing us to resolve luminal (CD24^+^CD29^low^) and myoepithelial (CD24^+^CD29^high^) populations. 98.24 ±0.4% of GFP^pos^ cells gated in MEC were found in the luminal subset by FACS analysis. Note that GFP^pos^ cells also display Tomato fluorescence 24 h after induction, as the Tomato protein is still present at this time point, even if recombination has occurred. Values are shown in average ± s.e.m, *n* = 6. (**D–E**) The expression of Notch1 in sorted luminal (CD24^+^CD29^low^) (LUM) and myoepithelial cells (CD24^+^CD29^high^) (MYO) mammary cells from 10-wk-old B6/N wild-type females was analyzed at the mRNA level by qRT-PCR in A and at the protein level by western blot in B. The relative mRNA expression was normalized to the housekeeping gene 18S in A, while lamin B1 was used as a loading control in E. For western blot analysis, we used anti-K8 and anti-K5 antibodies as controls for sorted luminal and myoepithelial cells, respectively. *n* = 2. **(F–G)** Representative sections of mammary ducts from 6-wk-old N1Cre^ERT2^R26^mTmG^ females show that GFP-expressing cells (in green) are invariably negative for ERα (in red in F) and PR expression (in red in G). DAPI stains DNA in blue, *n* = 3. Scale bars correspond to 20 µm in C–D and F–G and 10 µm in the insets.

Our observations during embryonic mammary development prompted us to use the Notch1 promoter to define stem cell hierarchies in the postnatal mammary gland. To this purpose, we examined Notch1-expressing cells in pubertal and adult N1Cre^ERT2^R26^mTmG^ mice 24 h after tamoxifen administration. Surprisingly, GFP-marked cells could only be detected within the luminal cell layer, as they expressed the luminal marker (K8) but not the myoepithelial-specific cytokeratin K5 at all postnatal developmental stages (Fig. 2A,B and S3A Fig.). We also confirmed that GFP^pos^ cells were indeed luminal (CD24^+^CD29^low^) by flow cytometry analysis (Fig. 2C). Quantification by Fluorescence Activated Cell Sorting (FACS) revealed that Notch1-expressing GFP^pos^ cells represent 13.27 ±2.9% and 25.28 ±3.6% of the total luminal population in puberty and adulthood, respectively. Moreover, both Notch1 mRNA (Fig. 2D) and protein (Fig. 2E) were exclusively found in luminal mammary cells, isolated by FACS, confirming and extending previous reports [21]. Unexpectedly, we found that Notch1-expressing cells invariably lacked the expression of ERα and PR (Fig. 2F,G), and we confirmed these results by quantitative Real Time Polymerase Chain Reaction (qRT-PCR) in sorted GFP-labeled cells (S3B,C Fig.).

### ERα^neg^ Mammary Luminal Cells Clonally Expand and Self-Renew In Vivo

**Fig 3:**
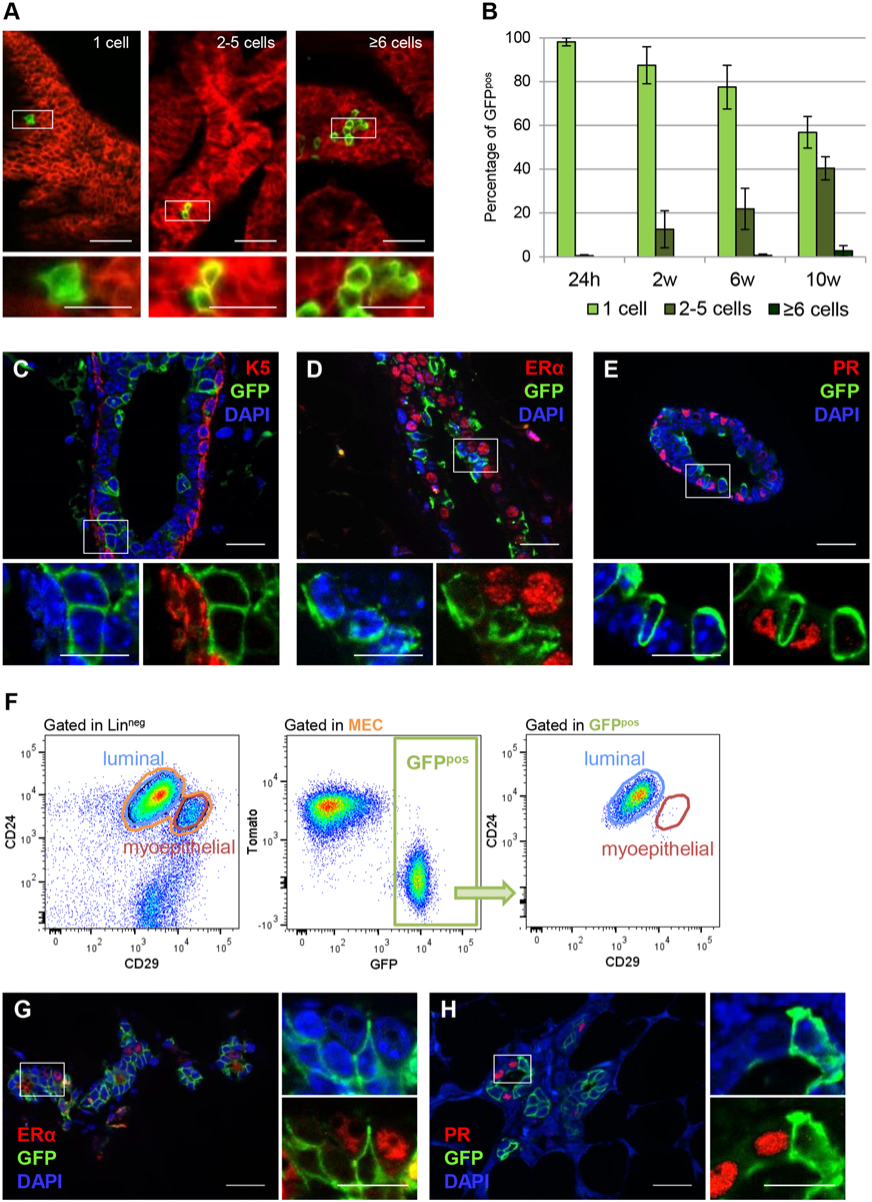
ERα^neg^ luminal cells represent self-sustained mammary progenitors that clonally expand and self-renew in vivo. **(A–B)** N1Cre^ERT2^R26^mTmG^ females induced with a low dose of tamoxifen (1µg/g of mouse body weight) at 4 wk of age and analyzed at different time points: 24 h (pulse), 2 wk (2 wk chase), 6 wk (6 wk chase), or 10 wk later (10 wk chase). **(A)** Representative images of whole mount digestion of mammary ducts labeling single cells (one cell), small clones (two to five cells), and large clones (six or more cells). Notch1-labelled cells are in green and Tomato fluorescence labels non-targeted cells. *n* = 11 mice. **(B)** Quantification of the in vivo clonal expansion of GFP-marked cells, at the indicated time points; *n* = 11. **(C–E)** N1Cre^ERT2^R26^mTmG^ females were induced with a single dose of tamoxifen (0.1mg/g of mouse body weight) at 4 wk of age and analyzed 10 wk later. The clonal progeny of Notch1-expressing cells (marked in green by GFP) is still luminal (K5^neg^ in red in C), and ERα^neg^ (in red in D) and PR^neg^ (in red in E), as the cells from which it derives. DAPI stains DNA in blue, *n* = 4. **(F)** FACS plots of N1Cre^ERT2^R26^mTmG^ females induced at puberty and analyzed after three cycles of pregnancy, lactation, and involution. Mammary epithelial cells (MEC) from females in the third week of the last involution were dissociated and analyzed by flow cytometry. GFP^pos^ cells represent 28.9 ±1.4% of the luminal population (CD24^+^and CD29^low^), *n* = 2. (**G–H**) Representative sections of mammary ducts from N1Cre^ERT2^R26^mTmG^ females induced at 4 wk of age and analyzed after three serial pregnancies. GFP-expressing cells (in green) remain invariably negative for ERα (in red in G) and PR expression (in red in H). DAPI stains DNA in blue, *n* = 2. Scale bars correspond to 40 µm in A; 20 µm in C–E, G and H; and 10 µm in the insets in C–E and G–H.

To examine the lineage potential of Notch1-expressing cells during pubertal mammary duct morphogenesis, we administered a reduced dose of tamoxifen (1µg/g of mouse body weight) to 4-wk-old females in order to target isolated single cells and analyzed the derived marked clones at different time points after tamoxifen injection. With this low dose of tamoxifen, we target only 0.074 ±0.008% of the mammary epithelial cells. To better quantify the clonal expansion of Notch1-marked cells, we classified GFP^pos^ clones as single cells, small clones (composed of two to five cells), or large clones (composed of six or more cells) (Fig. 3A), and we counted them at different time points (24 h, 2 wk, 6 wk, and 10 wk after induction). The quantification was performed on freshly dissected mammary glands that received a mild enzymatic digestion to remove the surrounding stroma but preserved intact fragments of ducts in 3-D, as we previously reported [14]. We observed that Notch1-expressing cells generate marked clones during pubertal development in vivo (Fig. 3A,B). The histological analysis of GFP-labeled progeny derived from Notch1-expressing cells after a 10 wk chase revealed that GFP^pos^ cells retain their luminal identity (Fig. 3C) and still remained ERα^neg^ and PR^neg^ (Fig. 3D,E), in sharp contrast with our findings in embryonically derived lineages (Fig. 1G).

To assess the self-renewal capacity of Notch1^pos^ luminal cells in vivo, we induced Cre recombination in pubertal females and analyzed them after three consecutive pregnancies and involutions. Remarkably, we found the same percentage of GFP^pos^ cells (28.9 ±1.4%) after three successive pregnancies (Fig. 3F), as in adult, virgin females, indicating that Notch1-expressing cells are maintained as a constant pool of ERα^neg^ and PR^neg^ cells (Fig. 3G,H) throughout mammary gland remodeling induced by pregnancy, and they represent cells able to survive involution.

Altogether, these results reveal that Notch1-expressing cells define ERα^neg^ mammary luminal cells as long-lived, unipotent stem cells, endowed with extensive self-renewal capacity. Importantly, our results are consistent with the existence of a common progenitor only during embryogenesis, while after birth the luminal ERα^pos^ and ER^neg^ cell populations maintain their own lineages independently.

### Notch1-Expressing Cells Represent ERα^neg^ Clonogenic Luminal Progenitors

**Fig 4:**
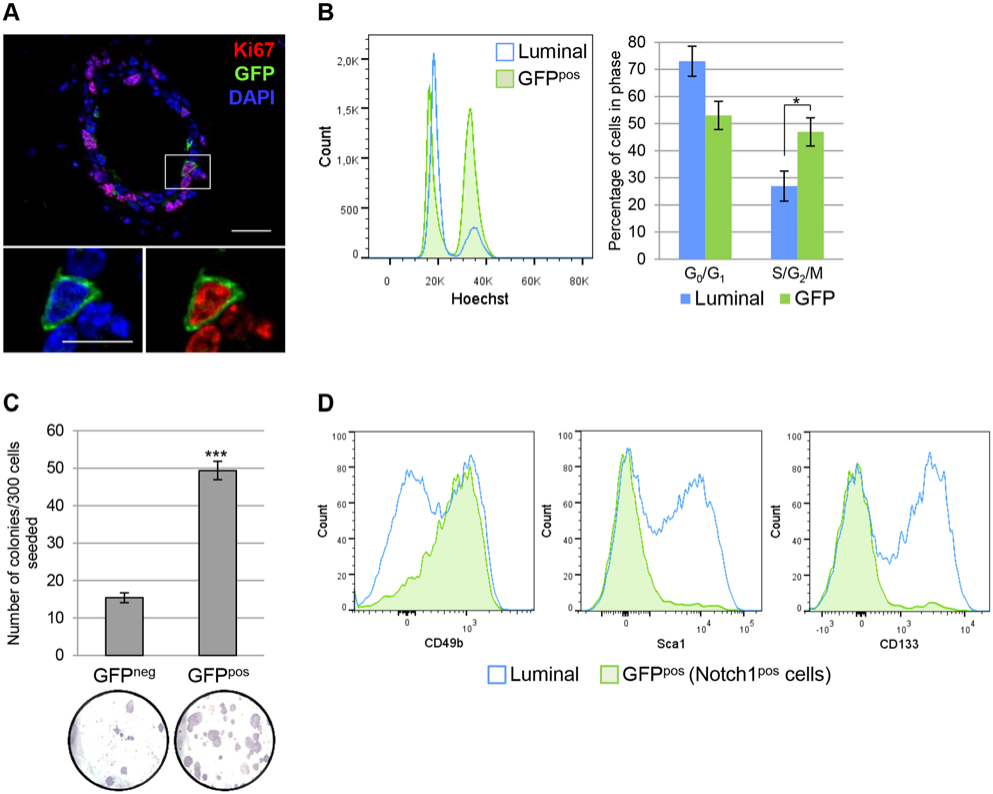
Notch1-expressing cells define highly clonogenic ERα^neg^ luminal progenitors. **(A)** N1Cre^ERT2^R26^mTmG^ females induced with tamoxifen at puberty (6 wk of age) and analyzed 24 h later present a high proportion of proliferative GFP^pos^ cells (73.45 ±3.72%), as indicated by Ki67 expression (in red); DAPI stains DNA in blue, *n* = 5. **(B)** Left, histogram of the cell cycle profile of total luminal cells (Luminal, in blue) compared to Notch1-expressing cells (GFP^pos^, in green) sorted by FACS and stained with Hoechst-33342 to evaluate their DNA content. Right, quantification of cycling (S/G2/M) and non-cycling (G_0_/G_1_) cells in total luminal cells (blue, S/G2/M = 26.9 ±5.5%) and in Notch1-expressing cells (green, S/G2/M = 47.0 ±5.2%). Data are represented as a mean ± s.e.m. of *n* = 5 animals, (*) *p* < 0.05 with *t* test. **(C)** N1Cre^ERT2^R26^mTmG^ females were induced with tamoxifen at 10 wk of age and analyzed 24 h later. GFP^neg^ and GFP^pos^ cells were sorted as Lin^-^CD24^+^CD29^low^ luminal cells by FACS, seeded on a feeder layer of irradiated fibroblast and their clonogenic capacity was evaluated after 7 d in culture. Top, quantification of the number of colonies generated by GFP^neg^ and GFP^pos^ cells. *n* = 3 experiments with two mice each, (***) *p* < 0.001 with *t* test. Bottom, representative pictures of counted colonies stained with Hematoxylin are shown below each bar. **(D)** Flow cytometry analysis indicates that GFP^pos^ cells (in green) are found within progenitor cell populations (CD49b^pos^, Sca1^neg^ and CD133^neg^) gated in the luminal population (Lin^-^CD24^+^CD29^low^) (in blue). GFP^pos^ cells represent 82.02 ±3.28% of CD49b^pos^ cells, 96.9 ±0.6% of Sca1^neg^ cells, and 93.7 ±1.0% of CD133^neg^ cells. *n* = 10. Scale bars correspond to 20 µm in A and 10 µm in the insets in A.

To determine if Notch1-expressing cells were actively cycling, we asked what percentage of GFP^pos^ cells was marked by the proliferation marker Ki67 and found that 73.45 ±3.72% of GFP^pos^ cells expressed Ki67 at puberty in 6-wk old mice (Fig. 4A). A quantitative analysis of cell cycle phases by FACS indicated that 46.9 ±5.1% of Notch1^pos^ cells were cycling (S-G_2_-M) compared to 26.9 ±5.5% of total luminal cells (Fig. 4B). In order to investigate the ability of Notch1-expressing cells to proliferate in vitro, we probed their clonogenic capacity compared to GFP^neg^ sorted luminal cells, when seeded on a feeder layer of irradiated 3T3 fibroblasts [28]. After 7 d in culture, GFP^pos^ cells had a strikingly higher colony-forming efficiency compared to GFP^neg^ luminal cells (Fig. 4C). These results indicate that the Notch1 receptor marks luminal ERα^neg^ progenitors with a high in vitro clonogenic potential. To further characterize these cells, we tested the expression of reported luminal progenitor markers in the GFP^pos^ population and found a remarkable overlap between Notch1^pos^ cells and luminal progenitors defined by the expression of CD49b [8]. Importantly, GFP^pos^ cells also lack expression of Sca1 and CD133 (Fig. 4D) [7], normally associated with non-clonogenic cells, further confirming their definition as luminal progenitor cells. Interestingly, when we explored the colony-forming ability of specific luminal cell populations sorted using these reported markers, we found that the CD133^neg^ and CD49b^pos^ cell subsets contain a mixture of equally clonogenic progenitors (S4A Fig.) that can be either ERα^pos^ (i.e., Notch1^neg^ cells) or Notch1^pos^ (corresponding to ERα^neg^ cells) (S4B,C Fig.). The quantification of the levels of Notch1 and ERα (*Esr1*) mRNA in the different subpopulations revealed that GFP^neg^ cells include both ERα^pos^ progenitor cells (CD133^neg^ and CD49b^pos^) and some non-targeted Notch1^pos^ cells (which did not undergo Cre-mediated recombination, due to the mosaic nature of this mouse line) (S4D Fig.). These results indicate that Notch1 is expressed exclusively in ERα^neg^ luminal progenitors, defining a specific marker for this important cell population.

### The Transcriptomic Profile of Notch1-Expressing Mammary Cells Defines a Signature of Luminal Progenitors

**Fig 5:**
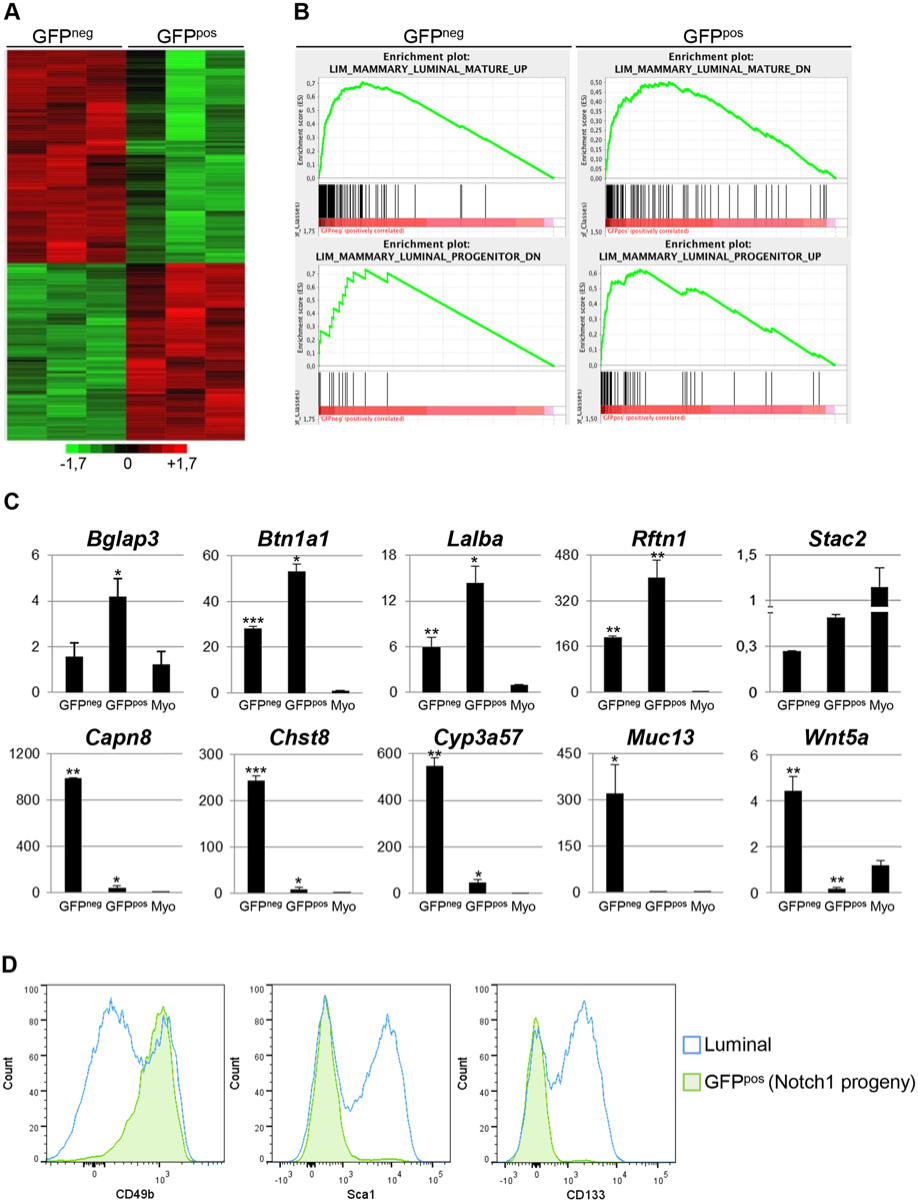
The transcriptional signature of ERα^neg^ mammary luminal progenitors is conserved in their derived lineages. **(A)** Genome-wide heat map showing the pattern of GFP^neg^ and GFP^pos^ luminal cells (Lin^-^CD24^+^CD29^low^) from 10-wk-old N1Cre^ERT2^R26^mTmG^ females induced for 24 h. Biological triplicates for each group were performed by pooling two females per experiment and are represented by different columns in the unsupervised clustering. **(B)** GSEA analysis showing the enrichment plots for GFP^neg^ and GFP^pos^ transcriptome profiles in four published gene signatures (Luminal_Mature_Up, Luminal_Mature_Down, Luminal_Progenitor_Up, Luminal_ Progenitor_Down). Sorted GFP^neg^ cells correlate with luminal mature cells, while sorted GFP^pos^ cells correspond to luminal progenitors. ES: Enrichment Score. **(C)** qRT-PCR analysis, in adult N1Cre^ERT2^R26^mTmG^ females induced for 24 h, of the top-ten ranked genes in the transcriptomic analysis (first row: up-regulated in GFP^pos^ and second row: down-regulated in GFP^pos^) confirms the differential expression of each of these genes detected in the microarray experiments. The expression levels of each gene in sorted myoepithelial cells are also shown (Myo). Relative mRNA expression was normalized to the housekeeping gene 18S. (*) *p* < 0.05, (**) *p* < 0.01, (***) *p* < 0.001 with *t* test. *n* = 2. **(D)** FACS analysis of sorted cells from N1Cre^ERT2^R26^mTmG^ females induced at 4 wk of age and analyzed 10 wk later (*n* = 8) shows that GFP^pos^ cells (Notch1-derived progeny, in green) overlap with ER^neg^ luminal progenitor markers (CD49b^pos^, Sca1^neg^ and CD133^neg^ in blue) gated as Lin^-^CD24^+^CD29^low^ luminal cells, showing an identical profile as Notch1-expressing cells 24 h post-induction (see Fig. 4D).

To gain further insights into the molecular signature characteristic of Notch1-expressing luminal progenitors, we performed a genome-wide microarray analysis comparing GFP^neg^ and GFP^pos^ sorted luminal cells (Fig. 5A and S1 Table). Gene Set Enrichment Analysis (GSEA) revealed that GFP^neg^ cells tightly correlate with a mature luminal signature, characteristic of terminally differentiated cells, whereas the expression profile of GFP^pos^ cells closely corresponds to a luminal progenitor signature (Fig. 5B) [29]. Moreover, we confirmed by qRT-PCR the differential expression of the top ten down-regulated and up-regulated genes that we obtained in the transcriptomic analysis, regardless of their putative function (Fig. 5C). These data define the complete transcriptional signature of ERα^neg^ luminal progenitors, which includes the expression of previously reported genes, such as Elf5, the Rank receptor (*Tnfrs11a*) [16], or milk-related proteins (*Ltf*, *Mfge8*) (S1 Table) [7]. We propose that the expression profile of Notch1-expressing ERα^neg^ cells could be relevant to identify important genes defining poorly differentiated basal-like breast tumors, believed to originate from ERα^neg^ luminal progenitor cells [5].

### ERα^neg^ Mammary Luminal Cells Are Self-Sustained Progenitors That Retain the Same Molecular Profile as the Cells They Derive From

Our findings indicated that Notch1 is expressed postnatally in a self-sustained population of luminal progenitors. When we characterized the identity of the Notch1-derived clonal progeny in adult mice, we were intrigued to observe that they expressed the same surface markers as the Notch1-expressing cells, even 10 wk after induction (Fig. 5D). To further confirm that Notch1-derived cells maintain the same molecular features as their “mothers,” we took an unbiased approach and assessed by qRT-PCR the expression levels of the top-ten ranking genes differentially expressed in GFP^neg^ and GFP^pos^ cells in our microarray data. These experiments indeed confirmed that Notch1-derived progeny 10 wk after CreERT2 induction retains an identical transcription profile as the cells analyzed after a 24 h tamoxifen pulse (S5 Fig.). Altogether, this compelling dataset unequivocally shows that Notch1-expressing progenitors exclusively give rise to cells with the same molecular profile in vivo in the postnatal mammary gland. These findings reveal the existence of specific unipotent stem cells that retain the memory of their cellular identity in vivo, ensuring their own long-term and faithful self-renewal, essential for proper tissue homeostasis.

### Notch1-Expressing Cells Designate Alveolar Progenitors at Pregnancy

**Fig 6:**
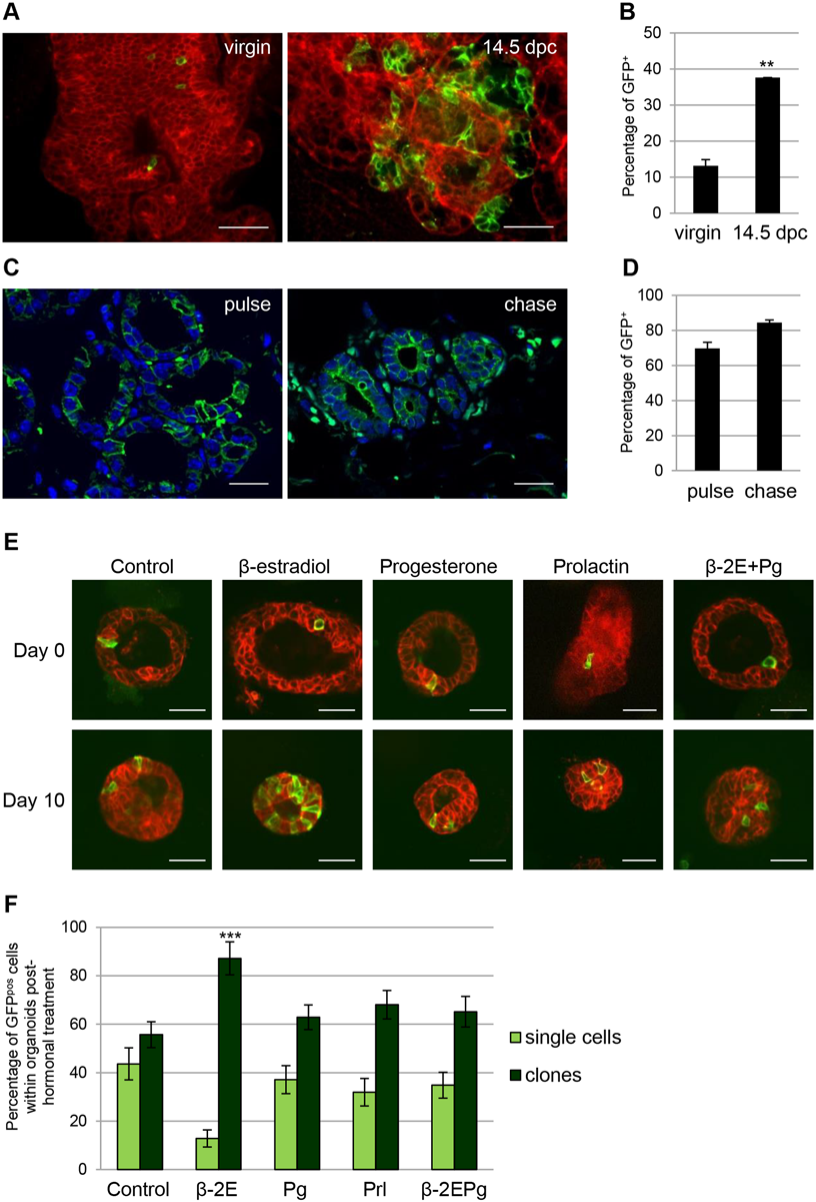
Notch1-expressing cells contribute to alveologenesis at pregnancy and expand in response to hormonal stimulation. **(A)** N1Cre^ERT2^R26^mTmG^ females were induced with a low dose of tamoxifen (1µg/g of mouse body weight) at 4 wk of age and analyzed as virgins (left) or at mid-pregnancy (14.5 d post coitum, dpc) (right panel). Representative pictures of whole mount mammary gland ducts are shown, where red denotes tomato fluorescence, while green indicates Notch1-derived GFP^pos^ cells. *n* = 3. **(B)** Quantification by flow cytometry of the percentage of GFP^pos^ luminal cells (Lin^-^CD24^+^CD29^low^) from N1Cre^ERT2^R26^mTmG^ females induced at 4 wk of age (0.1mg/g of mouse body weight) and analyzed at virgin or pregnant state (14.5 dpc). An extensive increase in the amount of GFP^pos^ cells is evident at pregnancy in A and B. *n* = 7. (**) *p* = 0.001 with *t* test. **(C)** Left, representative mammary section of N1Cre^ERT2^R26^mTmG^ females injected with tamoxifen at mid-pregnancy (14.5 dpc) and analyzed 24 h later (pulse). Right, mammary section of N1Cre^ERT2^R26^mTmG^ female injected at 4 wk of age and analyzed at mid-pregnancy (chase). **(D)** Quantification by flow cytometry of the percentage of GFP^pos^ cells luminal cells (Lin^-^CD24^+^CD29^low^) from N1Cre^ERT2^R26^mTmG^ females injected at mid-pregnancy (pulse: 69.75 ±3.55%) compared to females injected at 4 wk of age and analyzed at mid-pregnancy (chase: 86.1 ±2.01%). Values are shown as percentages ±s.e.m. of *n* = 5. **(E)** 3-D organotypic cultures from adult N1Cre^ERT2^R26^mTmG^ females were induced with 4-hydroxytamoxifen (4-OHT) in vitro and treated for 10 d with different hormones, as indicated. Red denotes tomato fluorescence and green indicates Notch1-marked cells at Day 0 and progeny at Day 10; *n* = 4. **(F)** Quantification of the clonal expansion of GFP^pos^ cells in 3-D organoids, classified as single cells (one cell, in light green) or clones (two or more cells, in dark green) indicates a significant expansion only in response to estradiol (β-2E bars). (***) *p* < 0.001 with *t* test. Scale bars correspond to 40 µm in A and E and 20 µm in C.

The observation that Notch1^pos^ cells are highly proliferating during puberty (Fig. 4A,B) but not in the adult gland (S6A Fig.), prompted us to ask if the proliferative behavior of these cells was influenced by circulating hormones, as would be predicted for ER^neg^ hormone-responding cells. Pregnancy triggers a wave of hormones, and we tested whether the cells marked by Notch1 in our mice would represent alveolar progenitors that expand extensively during pregnancy and contribute to the formation of alveolar buds. To this end, we labeled single cells with a low dose of tamoxifen (1µg/g of mouse body weight) in pubertal females and examined their clonal expansion at mid-pregnancy (14.5 dpc), when high levels of circulating hormones are released and the mammary gland undergoes dramatic remodeling. Pointing to a prominent role in alveologenesis, we observed that Notch1^pos^ cells massively expanded in pregnant mice compared to virgin females of the same age and they gave rise to GFP-marked alveoli (Fig. 6A,B). Importantly, we confirmed that even at this stage, Notch1-expressing cells only gave rise to Notch1-expressing luminal cells (S6B Fig.), as the percentage of GFP^pos^ cells at mid-pregnancy is comparable after a 24 h tamoxifen pulse and after a 10 wk chase (Fig. 6C,D). These results reveal that Notch1^pos^ luminal cells represent the alveolar progenitors of the mammary gland, responsible for the formation of milk-producing units during lactation. Of note, Notch1^pos^ cells are reminiscent of the recently characterized steroid receptor negative alveolar progenitors found in pregnancy and lactation as parity-identified mammary epithelial cells (PI-MECs) [30], but it is difficult at present to establish the relationship of the cells we describe here with PI-MECs in nulliparous females, as PI-MECs were only explored at pregnancy and lactation.

In order to better define the specific hormones to which Notch1^pos^ cells respond, we cultured fragments of mammary epithelium from adult mice as 3-D organotypic primary cultures [31] and examined the clonal growth of Notch1-expressing cells in the presence of three different hormones: β-estradiol (β-2E), progesterone (Pg) or prolactin (Prl). After 10 d in culture, we observed a net and prominent expansion of GFP^pos^ cells only in response to estrogen (β-estradiol) (Fig. 6E,F). To confirm that each exogenously added hormone efficiently activated its specific pathway, we assessed by qRT-PCR the expression of the immediate target genes activated in response to each hormonal treatment: progesterone receptor (*Pgr*) for estradiol signaling, Rank ligand (*Tnfs11*) for progesterone stimulation, and β-Casein (*Csn2*) for Prolactin [32–34]. A significantly elevated expression of mRNAs for each of these target genes, in response to stimulation with the corresponding hormone, clearly shows that cells grown as mammary organoids correctly respond to physiologic hormones (S6C Fig.). Of note, we could not detect any synergistic effect when we concomitantly treated the organoids with both estrogen and progesterone (Fig. 6E,F). These findings in ex vivo cultures, while not able to recapitulate the sequential waves of hormones that occur in vivo, corroborate the results we obtained in vivo during pregnancy and are consistent with the identification of Notch1-expressing cells as the estrogen-responsive cells of the mammary gland, stimulated in a paracrine fashion by neighboring ERα^pos^ cells [17]. As further discussed below, the ability of these cells to respond to hormones has important clinical applications in pathological conditions, as most current breast cancer treatments involve hormonal inhibitors, and the capacity of a given tumor to respond to these therapies can determine patient prognosis.

### Notch1-Expressing ERα^neg^ Luminal Cells Display Extensive Regenerative Capacity in Transplantation Assays

**Fig 7:**
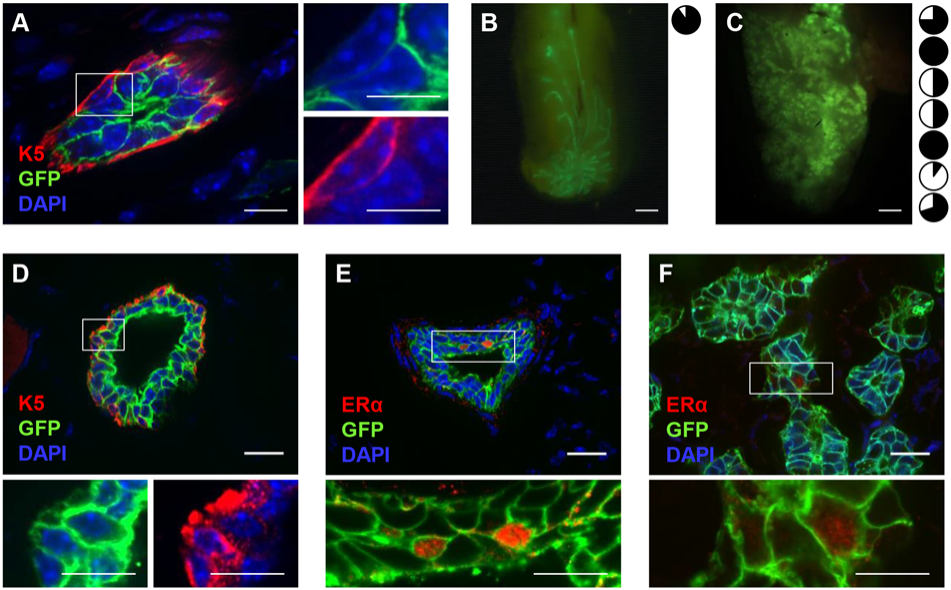
Notch1-expressing cells reveal a high regenerative capacity in transplantation assays. **(A)** 10,000 GFP^pos^ luminal cells were sorted by FACS within the CD24^+^/CD29^low^ gate, mixed 1:1 with Tomato epithelial cells (Lin^neg^), and injected in cleared fat pads of host mice. All transplanted animals showed outgrowths (*n* = 2). GFP^pos^ cells generated luminal cells but not myoepithelial cells, stained with anti-K5 antibody (in red in A). (**B–C**) Whole-mounts of GFP^pos^ outgrowths obtained in virgin host mice (*n* = 1 out of 7 transplanted, in B) and pregnant host females (*n* = 7 out of 11 transplanted, in C). On the right of each panel, graphic representations of the extent to which each transplant filled the fat pad (in black). **(D–F)** 10,000 sorted luminal GFP^pos^ (Notch1-expressing cells) cells were injected per fat pad, in the absence of myoepithelial cells. Immunofluorescence of representative sections of obtained outgrowths show that GFP^pos^ cells generate all mammary lineages, both in virgin (D–E) and in pregnant recipient mice (F). Immunofluorescence labeling of myoepithelial cells (anti-K5 in red in D), and ERα^pos^ (in red in E and F) show that GFP-derived clones contain K5^pos^ myoepithelial cells, ERα^pos^ and ERα^neg^ luminal cells. Scale bars correspond to 10 µm in A, 2 mm in B–C, and 20 µm in D–F.

To further ascertain the plasticity of adult Notch1-labeled cells, we tested their mammary repopulating capacity in transplantation assays [1]. As luminal mammary cells are known to produce mammary outgrowths very inefficiently when transplanted in the absence of myoepithelial cells, we first co-transplanted a mixture of sorted Tomato-labeled epithelial cells (Lin^neg^) with luminal GFP-marked Notch1^pos^ cells at a ratio of 1:1 in cleared fat pads of immunodeficient host mice. In this experimental setting, 6 wk after transplantation, GFP^pos^ cells could grow and gave rise to luminal cells that included both ERα^pos^ and ERα^neg^ cells (Fig. 7A and S7 Fig.), while the myoepithelial layer was formed by Tomato cells (Fig. 7A), confirming previous reports [4]. These findings demonstrate that in these regenerative conditions, Notch1-labeled luminal progenitors reveal a multipotent capacity that cannot be observed in vivo in adult homeostasis. Based on these important results, we also assayed the mammary repopulating ability of sorted GFP^pos^ luminal cells when transplanted in the absence of any myoepithelial cell. Given that we found that Notch1-labelled cells undergo a considerable expansion at pregnancy (see Fig. 6), we tested whether the regenerative capacity of sorted ER^neg^ progenitors was affected by hormonal stimulation. While only one out of eight outgrowths was obtained in host virgin females transplanted with 10,000 sorted GFP^pos^ cells (Fig. 7B), when we mated the host mice to induce pregnancy, we significantly increased the mammary repopulating capacity of Notch1-expressing cells, which produced outgrowths in seven out of 11 transplants (Fig. 7C). Remarkably, immunofluorescence analysis of these outgrowths revealed that GFP^pos^ cells had generated all mammary lineages, including myoepithelial K5^pos^ cells (Fig. 7D) and both ERα^pos^ and ERα^neg^ cells (Fig. 7E,F). These important results reveal the high plasticity of ERα^neg^ luminal progenitors and demonstrate that these cells have the ability of generating multiple lineages, although in adult homeostatic conditions they never show multipotency.

## Discussion

The cellular hierarchies between different luminal cell populations in the mammary gland in physiological conditions, as well as the molecular identity and dynamic in vivo behavior of stem and progenitor cells in this rather unique tissue are still not well understood. In this study we identified self-renewing ER^neg^ stem cells within the luminal mammary epithelium, able to maintain their precise cellular identity during development and tissue remodeling in pregnancy and involution, suggestive of a tight and long-term genetic and epigenetic memory.

Of relevance, the vast majority of breast cancers are thought to arise from luminal progenitors [5] and, as a consequence, the elucidation of the cellular hierarchy of this epithelial compartment in mammary development and adult homeostasis is fundamental to the eventual identification of the cells of origin of different breast tumor subtypes.

Despite the abundance of surface markers proposed to identify mammary cells with clonogenic capacity, the paucity of in vivo lineage tracing experiments has prevented a complete understanding of the developmental fate of these clonogenic cells and their contribution to homeostatic maintenance of the mammary gland. Proteins found to be specifically expressed in clonogenic cells have undeniably helped to discriminate between progenitors and mature luminal cells, but we still do not appreciate their in vivo behavior or the progeny that they physiologically produce at different developmental stages.

Through lineage-tracing studies using the Notch1 promoter, our results reveal the existence of ERα^neg^ luminal progenitors that are multipotent during embryonic development and become unipotent after birth (as they never generate myoepithelial nor ERα^pos^ cells in adult mice). These cells have extensive self-renewal capacity, they preserve their identity throughout adult life, and are highly responsive to hormones. Importantly, they can repopulate the entire mammary gland in transplantation assays, especially when they are stimulated by pregnancy-induced hormones.

Notably, our results evoke the involvement of a fundamental signaling pathway, like Notch, in retaining long-term cellular memory. In this context, is it important to mention that previous lineage tracing studies on Notch2-labelled [13] and Notch3-expressing [14] mammary cells have indicated that, while all these three Notch receptor paralogues are exclusively expressed in the luminal compartment, they seem to define distinct cell types. Indeed, mammary cells labeled by Notch1, Notch2, and Notch3CreERT2^SAT^ mice present different molecular features, morphology, localization, and in vivo behavior, unraveling the complexity and diversity of luminal lineages [15].

**Fig 8:**
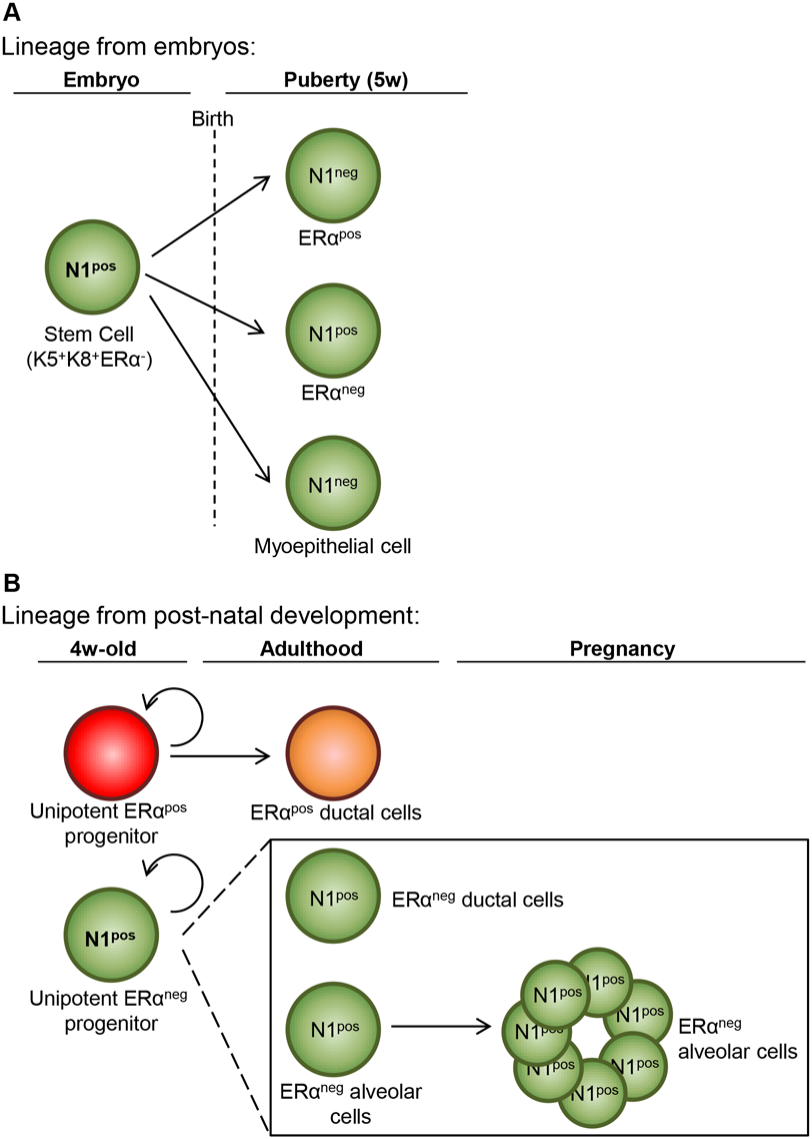
Proposed model for luminal cell hierarchy during mammary gland development. **(A)** Lineage tracing from embryos reveals that Notch1-expressing (N1^pos^) multipotent stem cells exist only during embryonic mammary development, when they co-express myoepithelial cytokeratin (K5) and luminal cytokeratin (K8). These multipotent stem cells can generate all mammary lineages (labeled in green postnatally). **(B)** After birth, luminal stem cells resolve in two distinct luminal progenitors: ERα^pos^ and ERα^neg^ cells, which maintain exclusively their own lineage throughout adulthood. Unipotent progenitors lacking ERα expression (ERα^neg^) marked by Notch1 are responsible for generating milk-producing alveoli at pregnancy and define self-renewing luminal cells able to survive mammary gland involution.

Our findings, that postnatally Notch1-expressing luminal progenitors can only give rise to identical progeny that maintains Notch1 expression, are completely unexpected and raise the question of the capacity of the rare ER^pos^ progenitors to produce different types of luminal cells. The results we obtained on the in vitro colony-forming potential of several luminal subsets indicate that the CD133^neg^ and CD49b^pos^ populations comprise two different progenitor subsets: ERα^neg^ (which would coincide with the Notch1-expressing cells characterized in this study) and ERα^pos^ progenitors (that would be Notch1^neg^) (S4 Fig.). The current absence of a mouse line able to exclusively label ER^pos^ luminal cells prevents a formal demonstration of the in vivo potential of these cells. However, we have previously reported that the Notch3 receptor was expressed at comparable frequency in both ER^pos^ and ER^neg^ luminal cells [14]. When we carefully analyzed the clones produced in vivo by single Notch3-expressing luminal cells, we found that the vast majority of Notch3-derived clones was composed of either exclusively ER^pos^ or ER^neg^ cells (S8 Fig.). These results are consistent with a model in which ER^pos^ and ER^neg^ progenitors can only give rise to ER^pos^ or ER^neg^ daughter cells, respectively, suggesting that these two populations of adult luminal progenitors define distinct, self-sustaining lineages (Fig. 8).

In this context, it is conceivable that embryonic Notch1-expressing could also represent distinct progenitors that would give rise to the different lineages observed postnatally. Indeed, while clonal tracing experiments in embryos show that Notch1-labeled embryonic cells can generate clones containing both myoepithelial and luminal ER^pos^ and ER^neg^ cells, it is difficult to rigorously determine if cells labeled with a single fluorophore are indeed part of the same clone or if they belong to two adjacent clones. Our results imply that embryonic mammary cells labeled by Notch1 expression can give rise to both myoepithelial and luminal lineages, but if these progenitors represent a unique cell type or distinct ones that co-express K5 and K8 remains to be established.

Our observations can have important implications for defining the cellular origin of specific breast cancer types. The possibility that a tumor may derive from an ER^pos^ or ER^neg^ luminal progenitor may dictate its molecular subtype, its growth characteristics and hormonal dependency, as well as its prognosis. Indeed, Molyneux and colleagues found that ER^neg^ luminal progenitors, and not basal stem cells, when targeted with a Brca1 mutation in mice, can yield mammary tumors that closely resemble human *BRCA1* mutant (basal-like) breast cancer, at least by histological examination [5]. In this context, it is noteworthy that, while several studies have reported a penetrant oncogenic effect of constitutively active forms of Notch1 in mammary tumorigenesis [21,35–37], the cells from which these tumors originate are not known, as all previous reports have used MMTV-driven transgenes, targeting the whole mammary epithelium. It will be interesting to use the Notch1-CreERT2^SAT^ mice described here to specifically induce oncogenic mutations in ERα^neg^ luminal progenitors and determine if this can influence the tumor outcome. Our results indicating that Notch1-expressing cells are extremely sensitive to hormonal stimulation and require estrogen to proliferate may partly explain why ectopic Notch activation in the mammary gland results in mammary tumors only upon pregnancy, suggesting that the cells we have characterized in this study may be linked to the increase in human breast cancer found to be associated with pregnancy and may be at the origin of hormonal-dependent human breast cancer subtypes.

## Materials and Methods

### Ethics Statement

All studies and procedures involving animals were in strict accordance with the European and National Regulation for the Protection of Vertebrate Animals used for Experimental and other Scientific Purposes (facility license #C75–05–18). We comply also with internationally established principles of replacement, reduction, and refinement in accordance with the Guide for the Care and Use of Laboratory Animals (NRC 2011). Husbandry, supply of animals, as well as maintenance and care of the animals in Exempt Of Pathogen Species (EOPS) environments before and during experiments fully satisfied the animal’s needs and welfare. Suffering of the animals has been kept to a minimum; no procedures inflicting pain have been performed.

### Mice

The generation of Notch1-CreERT2^SAT^ knock-in mice has been previously described [23]. These mice were crossed to a double fluorescent reporter mouse strain Rosa26^mTomato/mGFP^ [24], that was obtained by the Jackson Laboratories. For the chase experiments at E15.5, pregnant females were induced with tamoxifen and the embryos were extracted by C-sections at E19.5 and adopted by foster mothers, due to delivery failure caused by tamoxifen. All mice were injected with 0.1mg/g of mouse body weight of tamoxifen free base (Euromedex) as a standard dose or with 1µg/g of mouse body weight to label isolated cells where indicated in the text (also see S1 Fig.). For each experiment, four mammary glands of at least three mice were analyzed. No GFP expression was observed in non-induced mice.

### Mammary Gland Dissociation

Mammary glands were freshly dissected, harvested and placed into culture medium: DMEM/F12 GIBCO containing 1 mM glutamine, 5 mg/mL insulin (Sigma), 500 ng/mL hydrocortisone (Sigma), 10 ng/mL epidermal growth factor (Gibco), and 20 ng/mL cholera toxin (Sigma) and incubated with 600 U/mL collagenase (Sigma) and 200 U/mL hyaluronidase (Sigma) for 1 h at 37°C. Cells were then dissociated sequentially in 0.25% trypsin-EDTA for 3 min, 5 mg/mL dispase (Roche) and 0.1 mg/mL DNase I (Sigma) for 5 min, then NH_4_Cl followed by filtration through a 40 µm cell strainer to obtain a single cell preparation for FACS.

### Flow Cytometry, Antibodies, and Cell Sorting

Single cells were labeled with the required antibodies for 20 min at 4°C, washed, and re-suspended in DMEM/F12 medium containing 10 Mm HEPES, 5% FBS, and 1 mg/mL DAPI before analysis. The antibodies used were: biotin anti-CD133 (1:100; BioLegend), PE/Cy7 anti-mouse CD24 (1:100, BioLegend), PerCP/Cy5.5 anti-mouse Sca-1 (1:100, BioLegend), AlexaFluor700 anti-mouse/rat CD29 (1:100, BioLegend), PE anti-mouse CD49b (1:50, BioLegend); lineage markers: APC anti-mouse CD31 (1:100, BioLegend), APC anti-mouse Ter119 (1:100, BioLegend), APC anti-mouse CD45 (1:100, BioLegend); APC/Cy7 Streptavidin; isotype controls: PE rat IgM (1:50; BioLegend), PerCP/Cy5.5 rat IgGa (1:100, BioLegend). Single live cells were gated by DAPI exclusion and analyzed or sorted on LSRII, FACS Vantage or FACS Aria flow cytometers. The purity of sorted populations was about 95%.

### Immunofluorescence and Antibodies

Freshly dissected mammary glands were fixed in 4% paraformaldehyde and embedded in paraffin, or in optimal cutting temperature (OCT) medium (VWR International) for the transplantation experiments. Then, 4 µm sections were de-waxed and rehydrated through xylene and a gradient of ethanol and subjected to antigen retrieval in boiling citrate buffer for 20 min. The tissue was then permeabilized with 0.3% Triton X-100; non-specific antibody binding was blocked with 5% FBS and 10% BSA, and then sequentially incubated with primary and secondary antibodies. Primary antibodies used: chicken anti-GFP (1:1000; Abcam), rabbit anti-K5 (1:500; Covance), mouse anti-K8 (1:500; Covance), rabbit anti-Ki67 (1:500; Abcam), rabbit anti-PR (1:800; SantaCruz), and mouse anti-ERα (1:500; Dako); mouse anti-p63 (1:500, Abcam) and mouse anti-K14 (1:100, Abcam). Fluorochrome-conjugated secondary antibodies included AlexaFluor 488-conjugated anti-chicken IgG, Cy3-conjugated anti-rabbit IgG, Cy3-conjugated anti-mouse IgG for paraffin sections and Cy5-conjugated anti-rabbit and anti-mouse IgG for frozen sections, to avoid fluorophore overlap with the red tdTomato signal, visible exclusively in frozen sections. All secondary antibodies were used at 1:1000 dilutions and were purchased from Molecular Probes and Jackson ImmunoResearch Laboratories, Inc. Sections were counterstained with DAPI (1mg/mL; Sigma).

### RNA Extraction and qRT-PCR Analysis

RNA extraction was performed using Qiagen kit according to the manufacturer’s instructions. cDNA synthesis was achieved with the Superscript III First-strand cDNA synthesis kit (Invitrogen). The primer sequences used in this work are listed in S2 Table.

### Colony-Forming Assay

These experiments were performed as previously reported [28]. Briefly, freshly sorted mammary epithelial cells were plated on a feeder layer of irradiated fibroblasts in 24-well plates containing DMEM medium (Invitrogen) supplemented with 1% FCS (Invitrogen), 1% vol/vol penicillin/streptomycin (Invitrogen), 5 µg/mL of insulin (Sigma), 10 ng/mL of EGF (Gibco), and 100 ng/mL of Cholera toxin (Sigma). After 7 d in culture, colonies were ﬁxed in methanol, stained with Hematoxylin, and counted under a stereoscope.

### Isolation and Culture of Primary Mammary Organoids

Mammary glands were bilaterally dissected, finely minced and incubated for 1 hour at 37°C in DMEM/F-12 medium (Gibco) supplemented with 600 U/ml of Collagenase (Sigma) and 200 U/mL of Hyaluronidase (Sigma) on a shaking plate at 200 rpm. After several washes in PBS, organoids were separated from single cells through four differential centrifugations (pulse to 1,500 rpm in 10 ml DMEM/F12), as previously described [31]. The final pellet was then re-suspended in Growth Factor Reduced 100% Matrigel (BD Biosciences), and plated in 8-well coverslip bottom chambers (Idibi) and kept for 20 min at 37°C before addition of culture medium DMEM-F12 supplemented with Selenium/Transferrin, FGF2 (Sigma) and 1% Penicillin/Streptomycin (Life Technologies). After 24 h, 5 nM 4-OHT (Sigma-Aldrich) was added for 6 h. For testing hormone responses, the medium was supplemented with 1 µg/mL of prolactin (Sigma), 25 ng/mL of progesterone (Sigma), or 50 nM β-estradiol (Sigma). Cells were cultured for 10 d, and then imaged using an inverted confocal Spinning Disk microscope.

### Whole Mount Digestion

Females induced with 1 µg/g of tamoxifen were sacrificed and their mammary glands were digested with collagenase and hyaluronidase enzymes for 1–1.5 h at 37°C, as previously described [14]. After washing in PBS, the remaining mammary ducts were fixed in 4% PFA for 15 min and imaged using an inverted confocal Spinning Disk microscope.

### Microscope Image Acquisition

For image acquisition of stained sections, we used an Upright Epifluorescence Microscope with Apotome (Zeiss) or an upright Spinning disk Confocal Microscope (Roper/Zeiss), both equipped with a CoolSnap HQ2 CCD camera. Images were captured using either Axiovision AC software (Zeiss) or with Metamorph. Mammary organoids and whole mount digested mammary glands were imaged using an Inverted Spinning disk Confocal Microscope (Roper/Nikon) equipped with a CCD CoolSnap HQ2 camera, and images were captured with the Metamorph software. All images were processed and assembled with ImageJ and Adobe Photoshop software.

### Microarray and GSEA Analysis

Microarray analysis was performed using Affymetrix Mouse Gene 2.1 arrays. For each condition, three biological replicates were performed, by pooling the mammary glands from two mice/replicate. cDNAs were synthesized from total RNA and hybridized by the Institut Curie Genomics Platform. Data normalization and analysis was performed using the R software (version 3.0.0) and Bioconductor packages. Arrays were normalized according to the RMA normalization procedure [38]. After normalization, only annotated probe sets were selected and then filtered to obtain one probe set per each gene (the most variant was selected). The comparison between the GFP^pos^ and GFP^neg^ cells was performed using the linear model method with *Limma* package. We used false discovery rate (FDR) to adjust for multiple testing. Genes presenting a log fold change >1.5 and *p*-values adjusted by the Benjamini and Hochberg method <0.05 were considered statistically significant. We used GSEA v2.2 to perform the gene-set enrichment analysis on different functional and/or distinguishing gene signatures [39]. Because of the small number of samples per condition (*n* = 3), normalized microarray expression data were analyzed using gene-set permutation. Gene sets C2 (curated gene sets) were used for the analysis. These gene sets were obtained from the MSigDB database v4.0 adjusted for mouse organism based on The Jackson Laboratory Human and Mouse Homology and provided by The Walter and Eliza Hall Institute of Medical Research (http://bioinf.wehi.edu.au/software/MSigDB). Microarray data are available in the ArrayExpress database (www.ebi.ac.uk/arrayexpress) under accession number E-MTAB-2897.

### Transplantations into Cleared Mammary Fat Pads

10,000 GPF^pos^ luminal sorted cells (Lin^neg^CD24^+^CD29^low^) alone or mixed with 10,000 Tomato cells (Lin^neg^) were re-suspended in 10 µl of matrigel (BD Biosciences) and injected into cleared fat pads of glands number 4 of 3-wk-old Balb/C nude females. Recipient mice were mated 2 wk after transplantation and were analyzed when fully pregnant. Recipient glands were dissected, analyzed by whole-mount, fixed, and embedded in OCT for further immunostaining. An outgrowth was defined as an epithelial structure comprising ducts and lobules and/or terminal end buds.

The numerical data used in all figures are included in S1 Data.

## Supporting Information

S1 Data All numerical data used to build histograms and for statistics in this study are included in S1 Data. (XLSX)

S1 Fig (Related to Materials and Methods). Schematic description of tamoxifen regime and time of dissection. Schematic diagrams showing the time of tamoxifen administration and the time of analysis of mice used to detect either Notch1-expressing cells (pulses in A) or Notch1-derived progeny (chases in B) in the different experiments presented in this study. Red lines indicate the time of tamoxifen injection, and red triangles, the time of analysis.
(TIF)

S2 Fig (Related to Fig. 1). Notch1-expressing embryonic cells give rise to all mammary cell types. Pregnant females were induced with tamoxifen to label their embryos at embryonic day E15.5 and double transgenic N1Cre^ERT2^R26^mTmG^ littermates were analyzed 24 h later **(A)** or 5 wk after birth (**B–D**). **(A)** Representative embryonic mammary bud sections show that Notch1-expressing cells (marked by GFP in green in Ab) co-express myoepithelial (K5, in cyan in Aa and Ad) and luminal markers (K8, in red in Ac and Ad); *n* = 2. A shows the same image with all merged colors. (**B–D**) Representative pubertal mammary gland sections show that Notch1-derived clones (in green) contain myoepithelial (p63^pos^ in red in B, and K14^pos^ in red in C) and luminal PR^pos^ and PR^neg^ cells (anti-PR labeling in red in D), *n* = 3. DAPI stains DNA in blue. Scale bars correspond to 20 µm in A–D and 10 µm in the insets. (**E**) FACS plots of dissociated mammary cells from 5-wk-old N1Cre^ERT2^R26^mTmG^ females induced at E15.5. Cells were gated as Lin^neg^ cells (CD45/CD31/Ter119)^neg^ and then as mammary epithelial cells (MEC in orange) using the CD24 and CD29 markers, allowing us to resolve luminal (CD24^+^CD29^low^) and myoepithelial (CD24^+^CD29^high^) populations. 55.95 ±2.95% of GFP^pos^ cells were gated as MEC, of which 84.76% were luminal and 15.24% were myoepithelial, *n* = 2. Values indicate average ± s.e.m.
(TIF)

S3 Fig (Related to Fig. 2). Notch1 expression is restricted to luminal cells. (**A**) Representative sections of ducts from N1Cre^ERT2^R26^mTmG^ females analyzed 24 h upon tamoxifen injection at different developmental stages: pre-puberty (4-wk-old) and adulthood (10-wk-old). Immunofluorescence was performed with anti-K5 antibodies (labeling myoepithelial cells in red), anti-K8 (marking luminal cells in red), as indicated in each panel, anti-GFP (to reveal Notch1-marked cells in green) and DAPI (nuclei in blue). **(B)** FACS plots showing the gating strategy used to sort GFP^neg^ and GFP^pos^ luminal cells. **(C)** qRT-PCR displaying the relative mRNA expression of *Gfp*, *Notch1*, *Esr1* (ERα) and *Pgr* (PR) in sorted GFP^neg^ and GFP^pos^ cells (*n* = 5). Fold change values were normalized to the housekeeping gene 18S. (*) *p* < 0.05 and (**) *p* < 0.01 with *t* test.
(TIF)

S4 Fig (Related to Fig. 4). The correlation between the clonogenic capacity and ERα expression of different luminal cell subsets reveals the existence of distinct luminal progenitors. Adult N1Cre^ERT2^R26^mTmG^ females were analyzed after a 24 h tamoxifen pulse. (**A**) Number of colonies obtained per 300 cells seeded on each well in clonogenic assays. The cell subsets sorted with each marker are indicated under each bar. These graphs show that CD49b^pos^ and CD133^neg^ cells have the same clonogenic capacity regardless if they are GFP^pos^ or GFP^neg^. *n* = 5 different experiments with two animals each. (***) *p* < 0.001 with *t* test. (**B–C**) qRT-PCR for the relative mRNA expression of *Notch1* and ERa (*Esr1*) in cell subsets resolved with the anti-CD133 (**B**) and the CD49b (**C**) antibodies, normalized to 18S expression. The levels of *Esr1* (ERα) inversely correlate with Notch1 expression. (*) *p* < 0.05 and (**) *p* < 0.01 with *t* test. (**D**) Schematic representation of sorted clonogenic populations: luminal clonogenic cells (CD49^pos^/CD133^neg^) include both Notch1-expressing cells (GFP^pos^/ERα^neg^ in green) and ERα^pos^ progenitors (in orange). The GFP^neg^ sorted cells contain both Notch1^neg^ (ERα^pos^, in orange) and Notch1^pos^ cells that were not targeted by Cre recombination (ERα^neg^ in light green) due to mosaicism of this line.
(TIF)

S5 Fig (Related to Fig. 5). The transcriptional signature of ERα^neg^ mammary luminal progenitors is conserved in their derived lineages. qRT-PCR analysis of sorted cells from N1Cre^ERT2^R26^mTmG^ females induced at 4 wk of age and analyzed 10 wk later (*n* = 2). The differential expression of the top-ten ranked genes in GFP^pos^ and GFP^neg^ cells obtained in the microarray experiments is maintained even in Notch1-derived lineages. All mRNA expression values are normalized to the housekeeping gene 18S. (*) *p* < 0.05, (**) *p* < 0.01, (***) *p* < 0.001 with *t* test.
(TIF)

S6 Fig (Related to Fig. 6). Notch1-expressing cells do not present a proliferative advantage in the adult resting mammary gland. N1Cre^ERT2^R26^mTmG^ adult virgin females were induced at 10 wk of age and analyzed 24 h later. Left, histogram showing superimposable cell cycle profiles between total luminal cells (blue) and Notch1-expressing cells (GFP^pos^, green) obtained by flow cytometry and measured with Hoechst-33342 staining. Right, quantification of cycling (S/G_2_/M) and non-cycling cells (G_0_/G_1_) in total luminal cells (blue, S/G_2_/M = 6.16 ±2.39%) and in GFP^pos^ cells (green, S/G_2_/M = 5.5 ±2.28%) confirms no differences in the two cell populations. Data are represented as a mean ± s.e.m of *n* = 5 mice. **(B)** Representative mammary sections of N1Cre^ERT2^R26^mTmG^ females injected with tamoxifen (0.1 mg/g of mouse body weight) at mid-pregnancy (14.5 dpc) and analyzed 24 h later (*n* = 4). Immunolabeling with anti-K5 antibodies (in red) shows no overlap with GFP-marked cells. **(C)** qRT-PCR analysis of expression of Progesterone receptor (*Pgr)*, Rank Ligand (*Tnfs11)* and β-casein (*Csn2)* in mammary organoids stimulated with the hormones shown in B and grown for 10 d. Each hormonal treatment resulted in the specific activation of the direct target genes for β-estradiol (β-2E), Progesterone (Pg), and Prolactin (Prl), respectively. (*) *p* < 0.05 with *t* test.
(TIF)

S7 Fig (Related to Fig. 7). Both ERα^pos^ and ERα^neg^ luminal cells derive from transplanted GFP^pos^ sorted cells. Immunofluorescence staining with anti-ERα (cyan or magenta, as indicated in each panel) of representative sections of outgrowths derived from co-transplantations of a 1:1 mixture of GFP^pos^ luminal sorted cells (CD24^+^ CD29^low^) with Tomato epithelial cells (Lin^neg^). **(A)** Both Tomato cells (in red) and GFP^pos^ cells (in green) contribute to the formation of outgrowths. In contrast to adult homeostasis, in transplantation experiments Notch1-expressing cells are able to produce both ERα^pos^ and ERα^neg^ luminal cells (Aa and Ab). DAPI stains DNA in blue in A. Scale bars correspond to 20 µm in A and 5 µm in the insets.
(TIF)

S8 Fig (Related to Discussions). ERα^pos^ and ERα^neg^ luminal cells derive from distinct progenitors in the postnatal mammary gland. Immunofluorescence staining with anti-ERα (in red) and anti-GFP (in green) antibodies of representative sections of mammary gland from Notch3-CreERT2^SAT^/R26^mTmG^ females induced at puberty (4–5-wk-old) and analyzed 5 wk later (*n* = 5). The vast majority of marked clones derived from Notch3-expressing cells contain either only ERα^pos^ or only ERα^neg^ cells, strongly suggesting that they represent two separate cell lineages in the adult mammary gland.
(TIF)

S1 Table (Related to Fig. 3). Genes differentially express in luminal GFP^neg^ vs GFP^pos^. List of genes that ≥1.7 log Fold Change (logFC) different between luminal GFP^neg^ and GFP^pos.^ LogFC values, represented by the average of three different FACS experiments with two 10-wk-old virgin females each, indicates the degree of expression on change (1 represents no change, <1 indicates repression, >1 overexpression of gene). The *p*-value is the significance value of the expression change observed. The left column includes the genes downregulated in the GFP^pos^ and the right column the genes were upregulated compared to GFP^neg^.
(DOCX)

S2 Table (Related to Experimental Procedures). Primer sequences used for RT-PCR analysis in this work.
(DOCX)

## Abbreviations

APC: Allophycocyanin1
Cy: Cyanin
DAPI: 4',6-Diamidino-2-Phenylindole
ERα: Estrogen Receptor α
FACS: Fluorescence Activated Cell Sorting
GFP: Green Fluorescent Protein
GSEA: Gene Set Enrichment Analysis
K5: Cytokeratin 5
K8: Cytokeratin 8
Lin: Lineage
MEC: Mammary Epithelial Cells
MaSC: mammary stem cells
N1: Notch1
N3: Notch3
4-OHT: 4-hydroxytamoxifen
PE: PhycoErythrin
PerCP: Peridinin chlorophyll protein
PR: Progesterone Receptor
qRT-PCR: quantitative Real Time Polymerase Chain Reaction
Sca1: Stem cell antigen-1
