# Supplementary figures and images for: Luminal Progenitors Restrict Their Lineage Potential during Mammary Gland Development
Source: PLoS Biol. 2015 Feb 17;13(2):e1002069. doi: 10.1371/journal.pbio.1002069 (PMC4331521; doi:10.1371/journal.pbio.1002069)

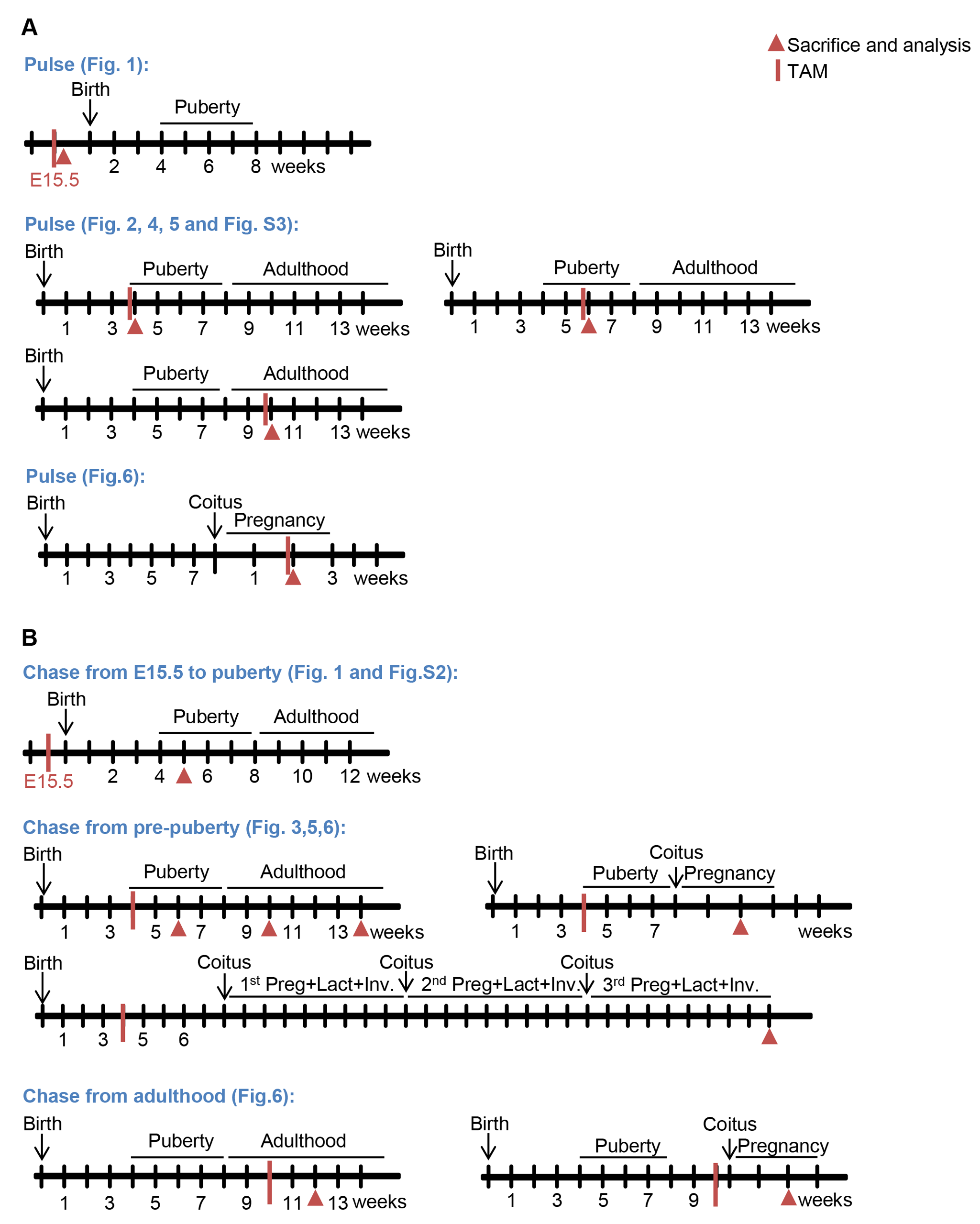

Supplement: S1 Fig — Schematic description of tamoxifen regime and time of dissection. Schematic diagrams showing the time of tamoxifen administration and the time of analysis of mice used to detect either Notch1-expressing cells (pulses in A) or Notch1-derived progeny (chases in B) in the different experiments presented in this study. Red lines indicate the time of tamoxifen injection, and red triangles, the time of analysis. (TIF) [file pbio.1002069.s002.tif]

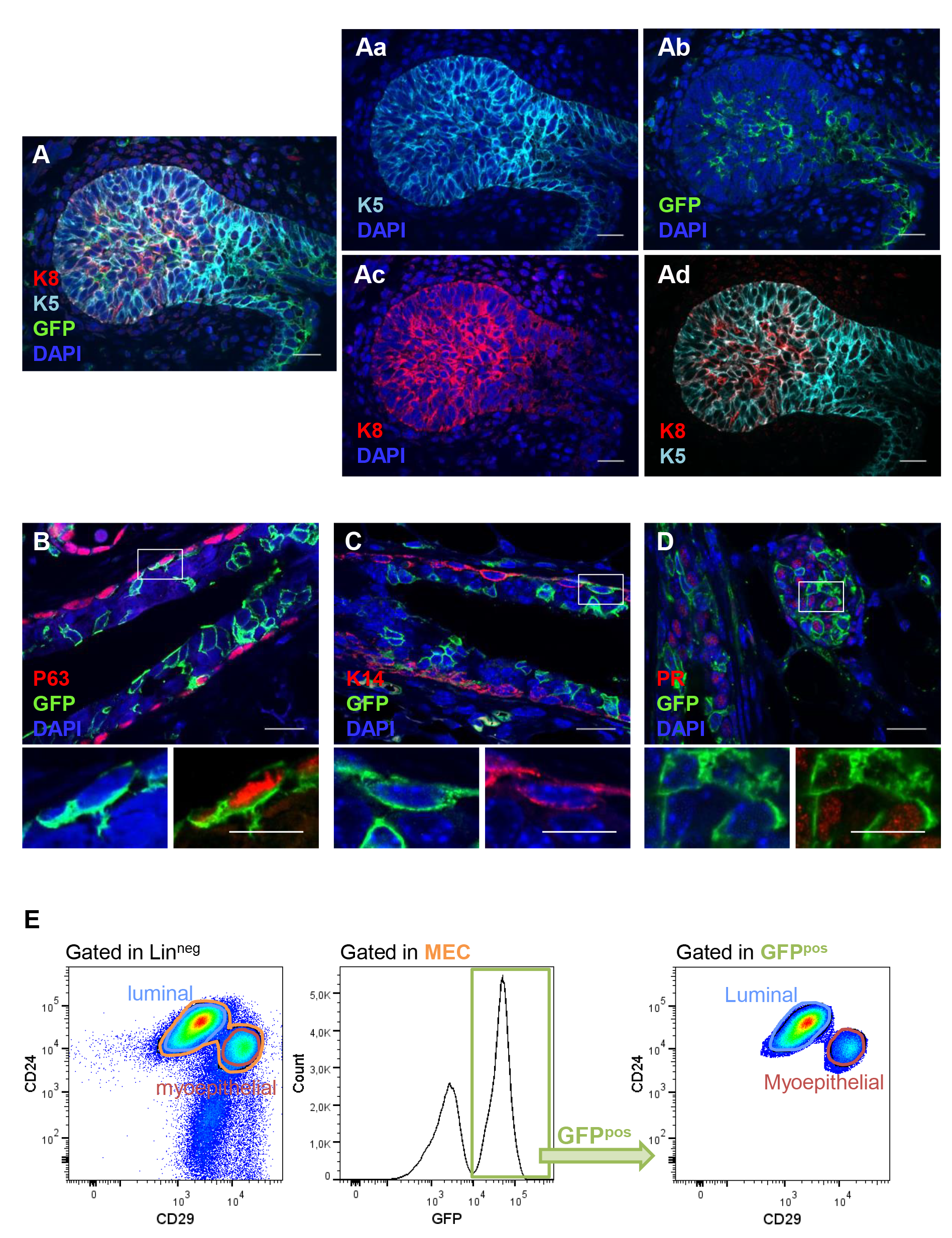

Supplement: S2 Fig — Notch1-expressing embryonic cells give rise to all mammary cell types. Pregnant females were induced with tamoxifen to label their embryos at embryonic day E15.5 and double transgenic N1CreERT2R26mTmG littermates were analyzed 24 h later (A) or 5 wk after birth (B–D). (A) Representative embryonic mammary bud sections show that Notch1-expressing cells (marked by GFP in green in Ab) co-express myoepithelial (K5, in cyan in Aa and Ad) and luminal markers (K8, in red in Ac and Ad); n = 2. A shows the same image with all merged colors. (B–D) Representative pubertal mammary gland sections show that Notch1-derived clones (in green) contain myoepithelial (p63pos in red in B, and K14pos in red in C) and luminal PRpos and PRneg cells (anti-PR labeling in red in D), n = 3. DAPI stains DNA in blue. Scale bars correspond to 20 µm in A–D and 10 µm in the insets. (E) FACS plots of dissociated mammary cells from 5-wk-old N1CreERT2R26mTmG females induced at E15.5. Cells were gated as Linneg cells (CD45/CD31/Ter119)neg and then as mammary epithelial cells (MEC in orange) using the CD24 and CD29 markers, allowing us to resolve luminal (CD24+CD29low) and myoepithelial (CD24+CD29high) populations. 55.95 ±2.95% of GFPpos cells were gated as MEC, of which 84.76% were luminal and 15.24% were myoepithelial, n = 2. Values indicate average ± s.e.m. (TIF) [file pbio.1002069.s003.tif]

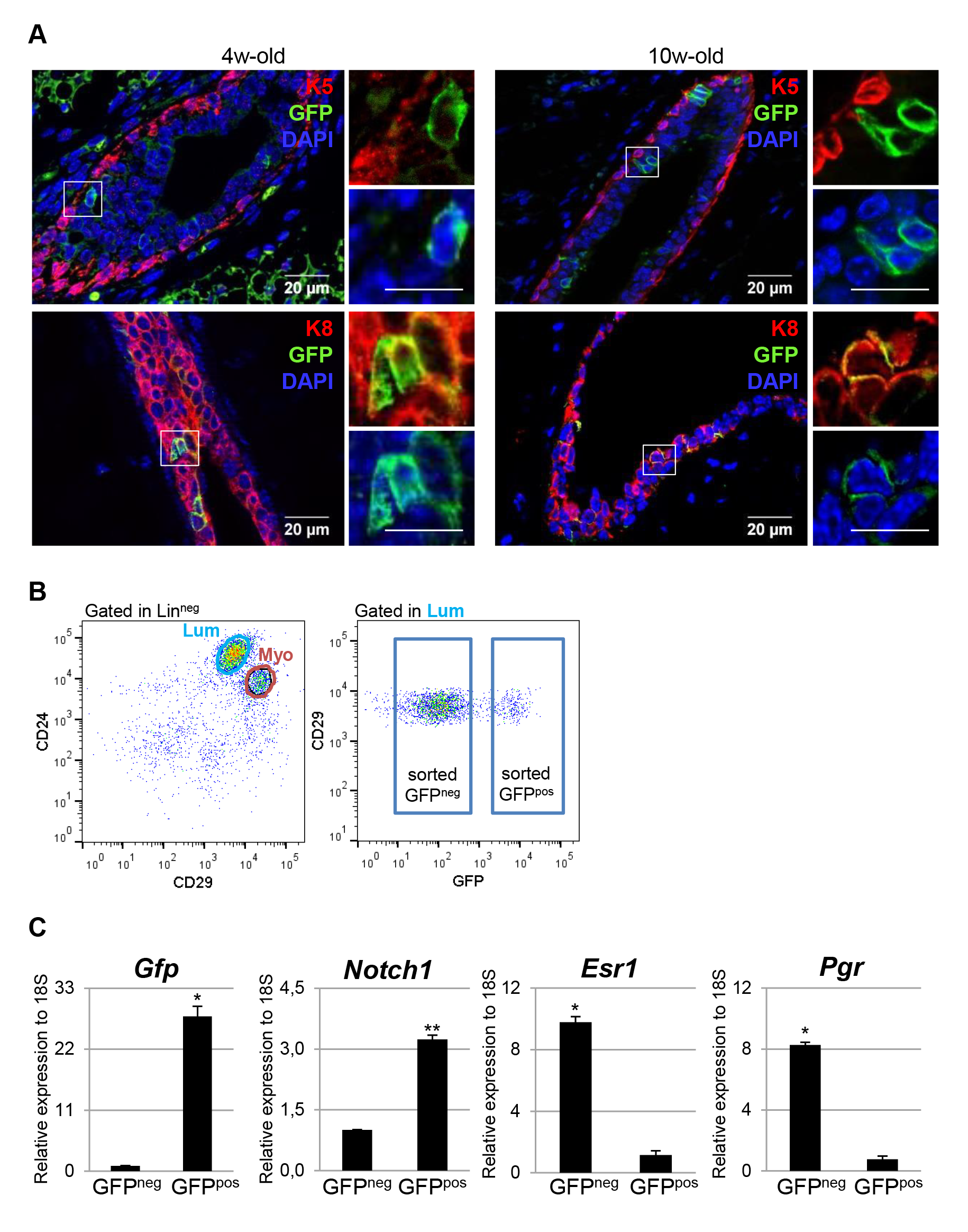

Supplement: S3 Fig — Notch1 expression is restricted to luminal cells. (A) Representative sections of ducts from N1CreERT2R26mTmG females analyzed 24 h upon tamoxifen injection at different developmental stages: pre-puberty (4-wk-old) and adulthood (10-wk-old). Immunofluorescence was performed with anti-K5 antibodies (labeling myoepithelial cells in red), anti-K8 (marking luminal cells in red), as indicated in each panel, anti-GFP (to reveal Notch1-marked cells in green) and DAPI (nuclei in blue). (B) FACS plots showing the gating strategy used to sort GFPneg and GFPpos luminal cells. (C) qRT-PCR displaying the relative mRNA expression of Gfp, Notch1, Esr1 (ERα) and Pgr (PR) in sorted GFPneg and GFPpos cells (n = 5). Fold change values were normalized to the housekeeping gene 18S. (*) p < 0.05 and (**) p < 0.01 with t test. (TIF) [file pbio.1002069.s004.tif]

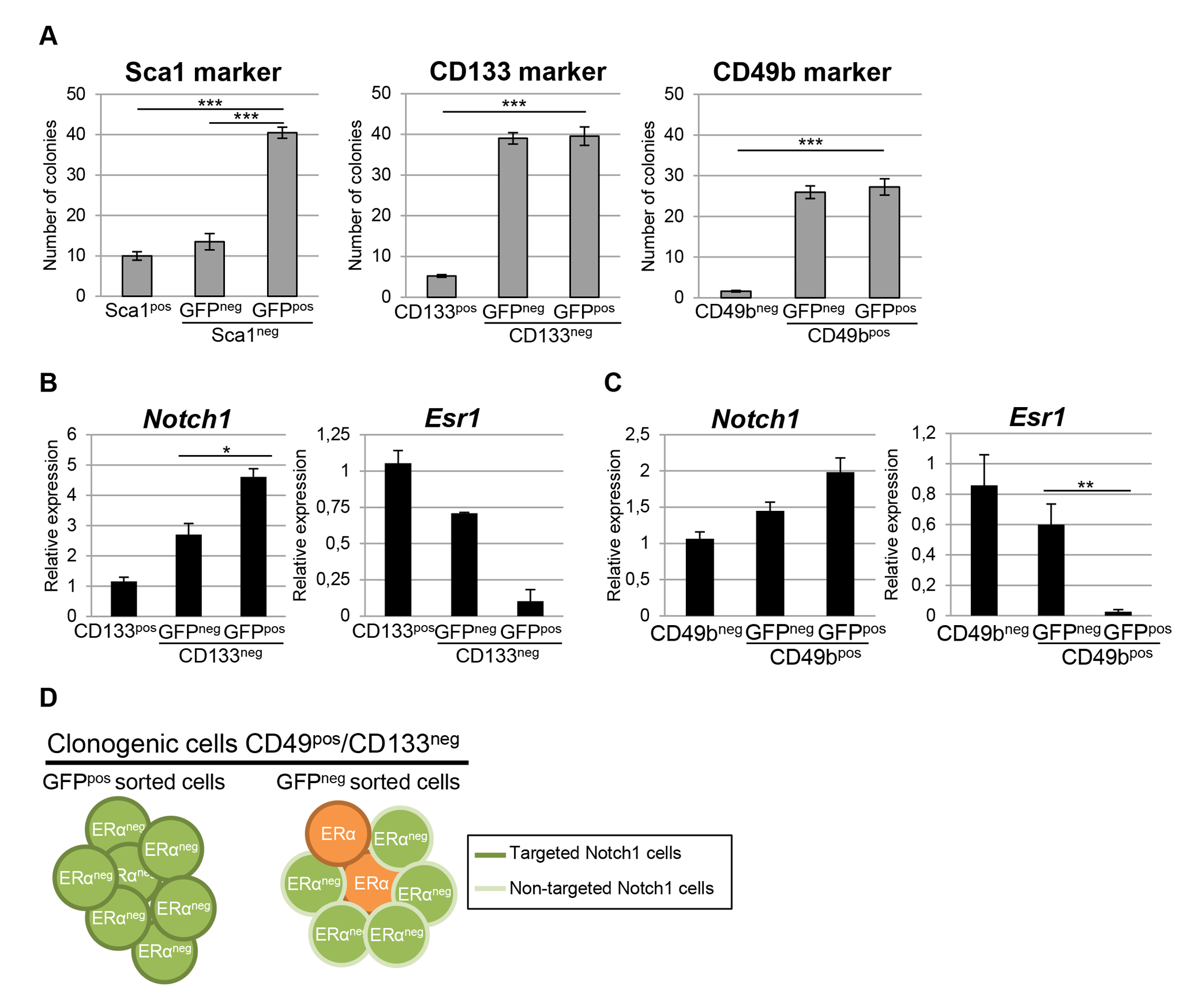

Supplement: S4 Fig — The correlation between the clonogenic capacity and ERα expression of different luminal cell subsets reveals the existence of distinct luminal progenitors. Adult N1CreERT2R26mTmG females were analyzed after a 24 h tamoxifen pulse. (A) Number of colonies obtained per 300 cells seeded on each well in clonogenic assays. The cell subsets sorted with each marker are indicated under each bar. These graphs show that CD49bpos and CD133neg cells have the same clonogenic capacity regardless if they are GFPpos or GFPneg. n = 5 different experiments with two animals each. (***) p < 0.001 with t test. (B–C) qRT-PCR for the relative mRNA expression of Notch1 and ERa (Esr1) in cell subsets resolved with the anti-CD133 (B) and the CD49b (C) antibodies, normalized to 18S expression. The levels of Esr1 (ERα) inversely correlate with Notch1 expression. (*) p < 0.05 and (**) p < 0.01 with t test. (D) Schematic representation of sorted clonogenic populations: luminal clonogenic cells (CD49pos/CD133neg) include both Notch1-expressing cells (GFPpos/ERαneg in green) and ERαpos progenitors (in orange). The GFPneg sorted cells contain both Notch1neg (ERαpos, in orange) and Notch1pos cells that were not targeted by Cre recombination (ERαneg in light green) due to mosaicism of this line. (TIF) [file pbio.1002069.s005.tif]

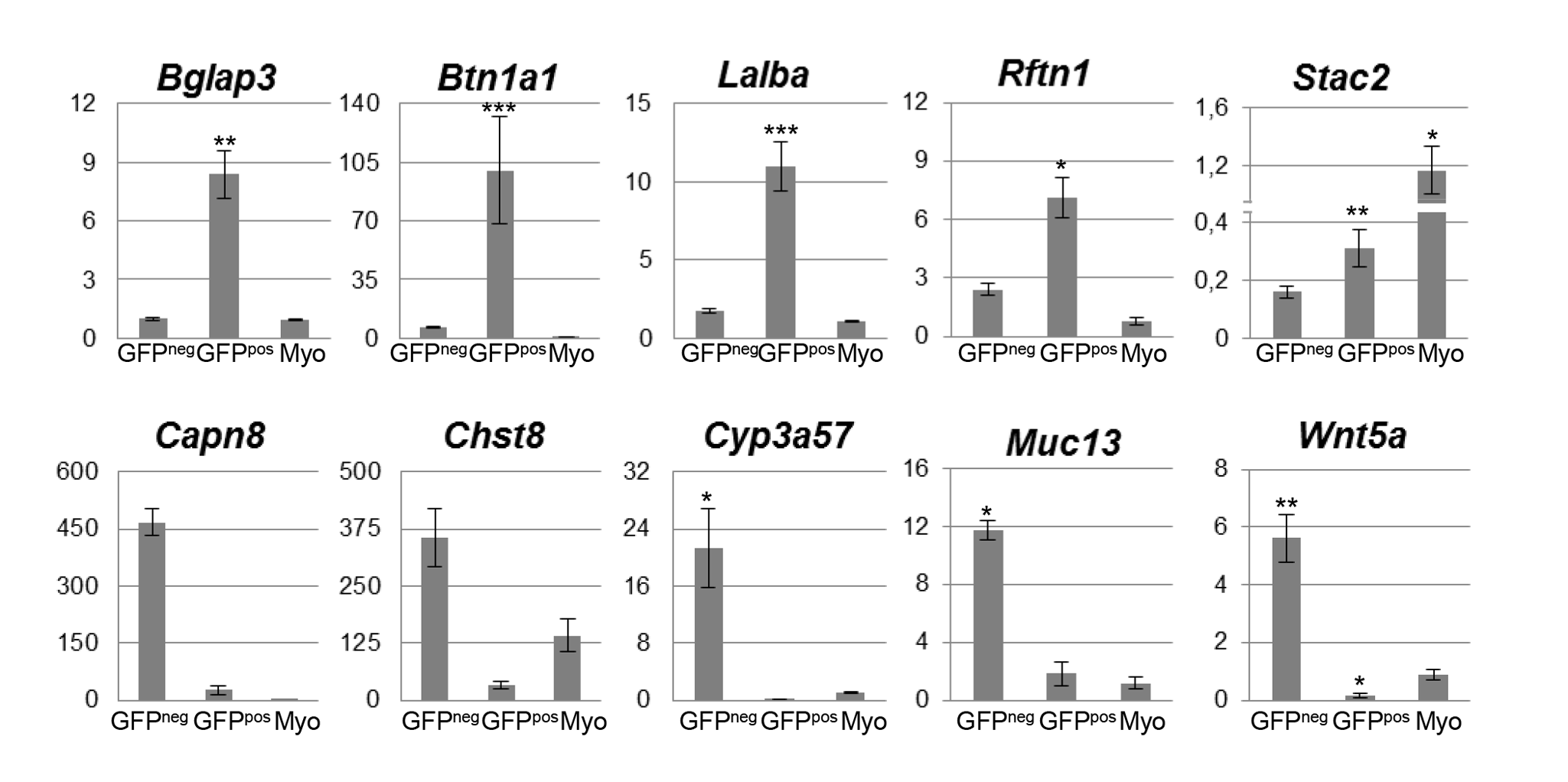

Supplement: S5 Fig — The transcriptional signature of ERαneg mammary luminal progenitors is conserved in their derived lineages. qRT-PCR analysis of sorted cells from N1CreERT2R26mTmG females induced at 4 wk of age and analyzed 10 wk later (n = 2). The differential expression of the top-ten ranked genes in GFPpos and GFPneg cells obtained in the microarray experiments is maintained even in Notch1-derived lineages. All mRNA expression values are normalized to the housekeeping gene 18S. (*) p < 0.05, (**) p < 0.01, (***) p < 0.001 with t test. (TIF) [file pbio.1002069.s006.tif]

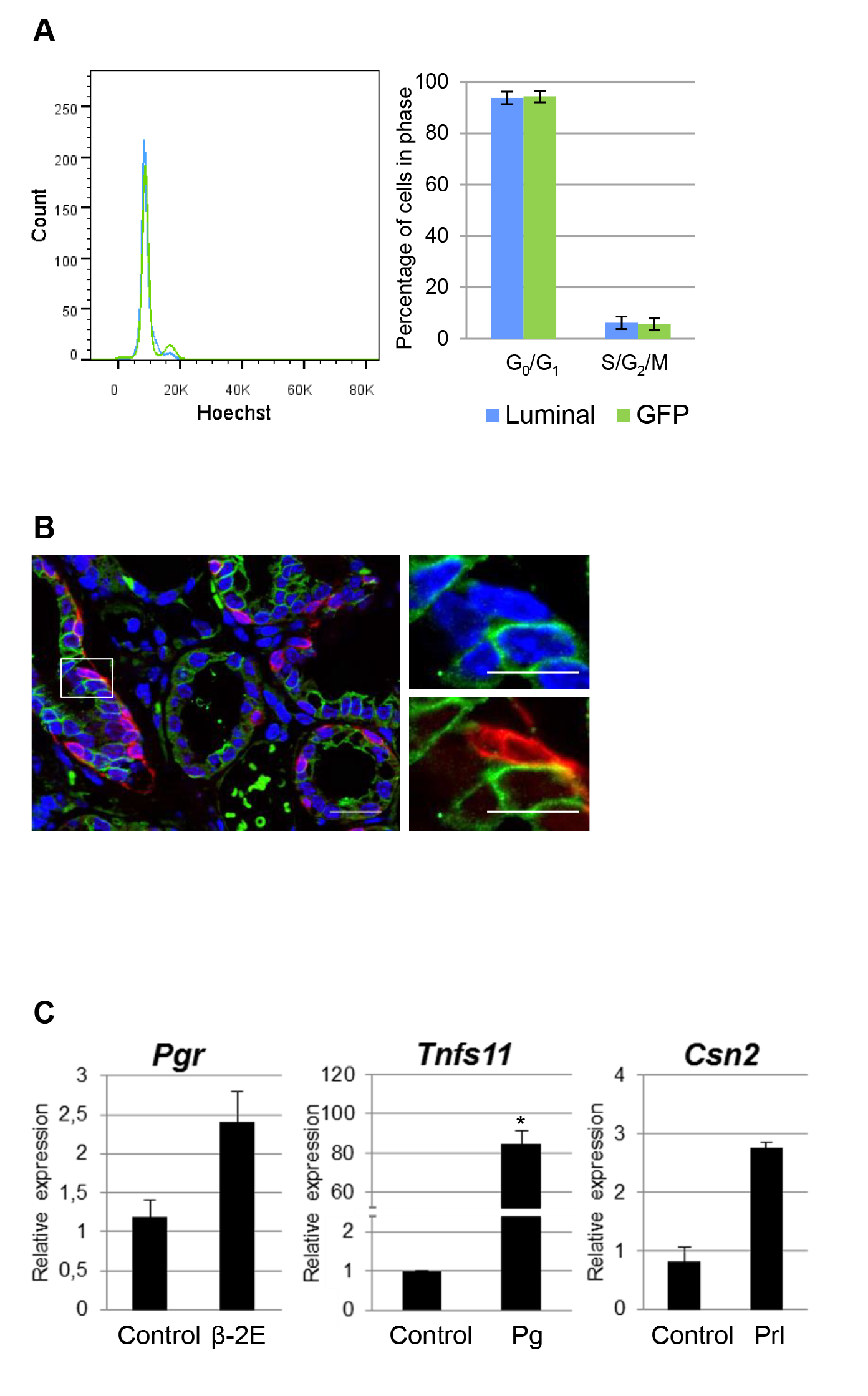

Supplement: S6 Fig — Notch1-expressing cells do not present a proliferative advantage in the adult resting mammary gland. N1CreERT2R26mTmG adult virgin females were induced at 10 wk of age and analyzed 24 h later. Left, histogram showing superimposable cell cycle profiles between total luminal cells (blue) and Notch1-expressing cells (GFPpos, green) obtained by flow cytometry and measured with Hoechst-33342 staining. Right, quantification of cycling (S/G2/M) and non-cycling cells (G0/G1) in total luminal cells (blue, S/G2/M = 6.16 ±2.39%) and in GFPpos cells (green, S/G2/M = 5.5 ±2.28%) confirms no differences in the two cell populations. Data are represented as a mean ± s.e.m of n = 5 mice. (B) Representative mammary sections of N1CreERT2R26mTmG females injected with tamoxifen (0.1 mg/g of mouse body weight) at mid-pregnancy (14.5 dpc) and analyzed 24 h later (n = 4). Immunolabeling with anti-K5 antibodies (in red) shows no overlap with GFP-marked cells. (C) qRT-PCR analysis of expression of Progesterone receptor (Pgr), Rank Ligand (Tnfs11) and β-casein (Csn2) in mammary organoids stimulated with the hormones shown in B and grown for 10 d. Each hormonal treatment resulted in the specific activation of the direct target genes for β-estradiol (β-2E), Progesterone (Pg), and Prolactin (Prl), respectively. (*) p < 0.05 with t test. (TIF) [file pbio.1002069.s007.tif]

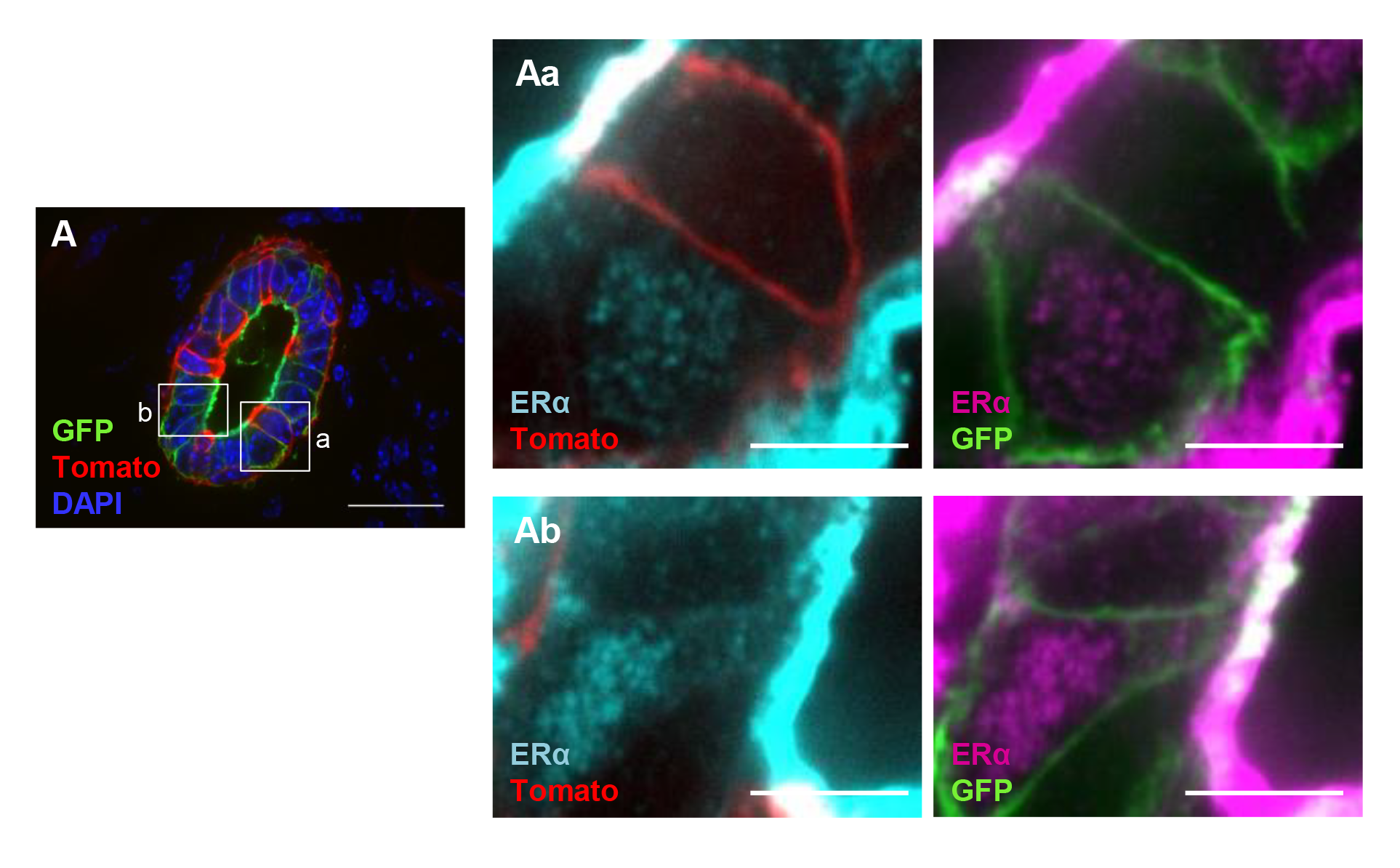

Supplement: S7 Fig — Both ERαpos and ERαneg luminal cells derive from transplanted GFPpos sorted cells. Immunofluorescence staining with anti-ERα (cyan or magenta, as indicated in each panel) of representative sections of outgrowths derived from co-transplantations of a 1:1 mixture of GFPpos luminal sorted cells (CD24+ CD29low) with Tomato epithelial cells (Linneg). (A) Both Tomato cells (in red) and GFPpos cells (in green) contribute to the formation of outgrowths. In contrast to adult homeostasis, in transplantation experiments Notch1-expressing cells are able to produce both ERαpos and ERαneg luminal cells (Aa and Ab). DAPI stains DNA in blue in A. Scale bars correspond to 20 µm in A and 5 µm in the insets. (TIF) [file pbio.1002069.s008.tif]

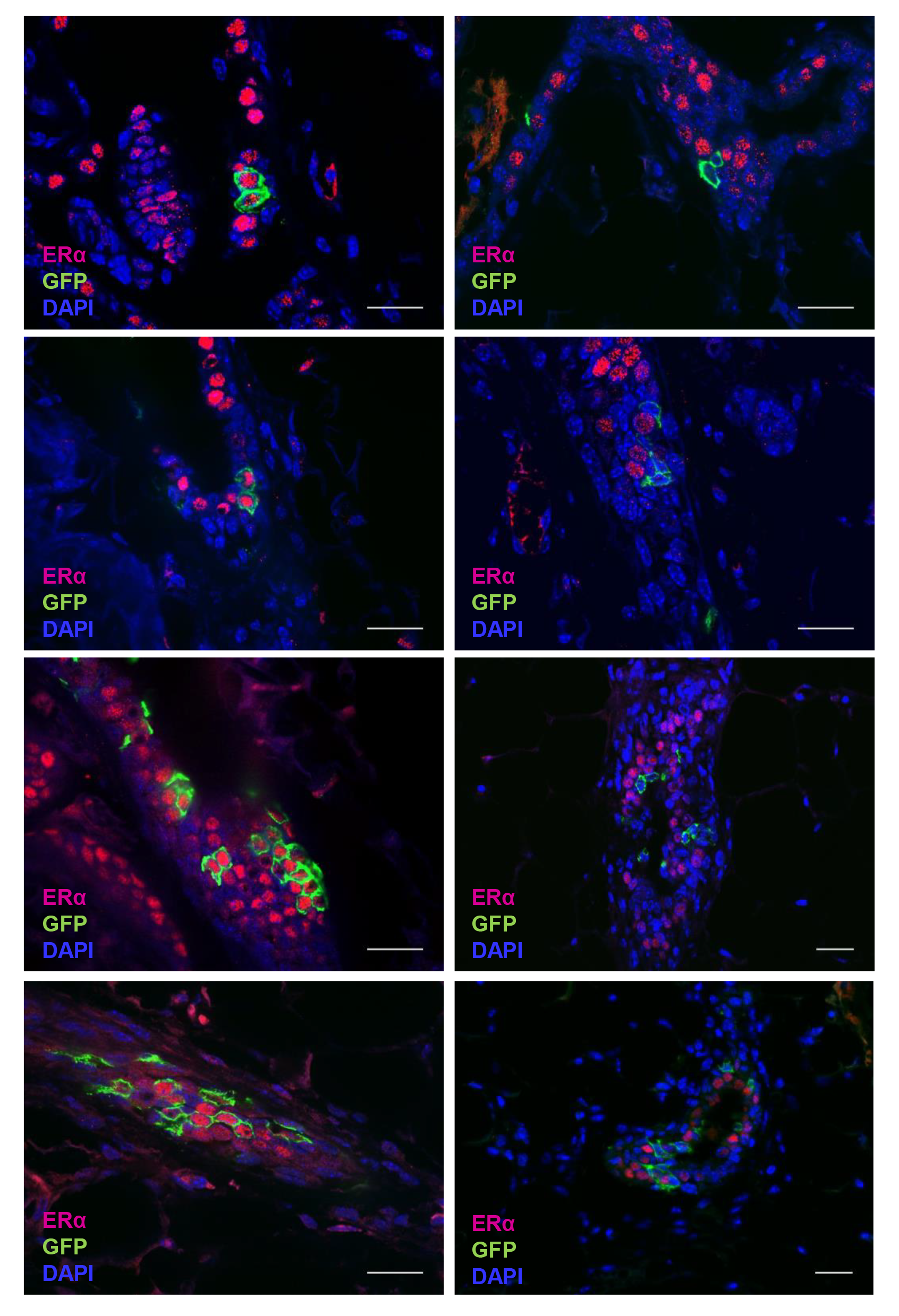

Supplement: S8 Fig — ERαpos and ERαneg luminal cells derive from distinct progenitors in the postnatal mammary gland. Immunofluorescence staining with anti-ERα (in red) and anti-GFP (in green) antibodies of representative sections of mammary gland from Notch3-CreERT2SAT/R26mTmG females induced at puberty (4–5-wk-old) and analyzed 5 wk later (n = 5). The vast majority of marked clones derived from Notch3-expressing cells contain either only ERαpos or only ERαneg cells, strongly suggesting that they represent two separate cell lineages in the adult mammary gland. (TIF) [file pbio.1002069.s009.tif]
