# Supplementary material for: Luminal Progenitors Restrict Their Lineage Potential during Mammary Gland Development
Source: PLoS Biol. 2015 Feb 17;13(2):e1002069. doi: 10.1371/journal.pbio.1002069 (PMC4331521; doi:10.1371/journal.pbio.1002069)
Supplement: S1 Table — Genes differentially express in luminal GFPneg vs GFPpos. List of genes that ≥1.7 log Fold Change (logFC) different between luminal GFPneg and GFPpos. LogFC values, represented by the average of three different FACS experiments with two 10-wk-old virgin females each, indicates the degree of expression on change (1 represents no change, <1 indicates repression, >1 overexpression of gene). The p-value is the significance value of the expression change observed. The left column includes the genes downregulated in the GFPpos and the right column the genes were upregulated compared to GFPneg. (DOCX) [file pbio.1002069.s010.docx]

**S1 Table. Genes differentially expressed in GFP^neg^ vs GFP^pos^ cells.**

| Gene Symbol | **logFC** | **p.value** | **Gene Symbol** | **logFC** | **p.value** |
| --- | --- | --- | --- | --- | --- |
| Cyp3a57 | -3,10 | 0,021 | **Lalba** | 3,09 | 0,022 |
| Mir5124 | -3,05 | 0,007 | **Stac2** | 2,64 | 0,006 |
| Gm16065 | -2,97 | 0,032 | **Bglap3** | 2,63 | 0,007 |
| Chst8 | -2,82 | 0,044 | **Rftn1** | 2,58 | 0,006 |
| Capn8 | -2,80 | 0,006 | **Btn1a1** | 2,56 | 0,010 |
| Wnt5a | -2,79 | 0,026 | **Srgap3** | 2,52 | 0,004 |
| Muc13 | -2,76 | 0,027 | **Nqo1** | 2,51 | 0,004 |
| R3hdml | -2,76 | 0,023 | **Ccdc129** | 2,50 | 0,005 |
| Fam43a | -2,75 | 0,047 | **Xdh** | 2,48 | 0,005 |
| Abcc8 | -2,73 | 0,010 | **Kcnn4** | 2,47 | 0,004 |
| Il18r1 | -2,72 | 0,032 | **Slc5a1** | 2,46 | 0,004 |
| Mtmr7 | -2,67 | 0,011 | **Fabp5** | 2,44 | 0,010 |
| BC030870 | -2,64 | 0,009 | **Efna5** | 2,44 | 0,010 |
| Gm6976 | -2,64 | 0,010 | **Sox10** | 2,43 | 0,005 |
| Col8a1 | -2,64 | 0,013 | **Itga8** | 2,41 | 0,005 |
| Kif5c | -2,64 | 0,021 | **Hhipl2** | 2,40 | 0,006 |
| Sytl5 | -2,63 | 0,024 | **Clmn** | 2,39 | 0,007 |
| Folr2 | -2,62 | 0,021 | **Itgb8** | 2,39 | 0,004 |
| Itih2 | -2,62 | 0,044 | **Tmtc1** | 2,37 | 0,005 |
| Gfpt2 | -2,61 | 0,020 | **Gprc5b** | 2,36 | 0,011 |
| Gm4951 | -2,60 | 0,010 | **Gm20317** | 2,35 | 0,030 |
| Usp18 | -2,58 | 0,018 | **Tiam2** | 2,35 | 0,004 |
| Prrt1 | -2,57 | 0,024 | **Nav3** | 2,34 | 0,007 |
| Mia2 | -2,56 | 0,015 | **2810468N07Rik** | 2,33 | 0,004 |
| Egflam | -2,55 | 0,010 | **Grik3** | 2,32 | 0,040 |
| Samd5 | -2,55 | 0,021 | **Gpc6** | 2,32 | 0,007 |
| Pon3 | -2,54 | 0,028 | **Acsl1** | 2,31 | 0,007 |
| Piezo2 | -2,53 | 0,015 | **Crispld2** | 2,30 | 0,005 |
| Tubg2 | -2,52 | 0,007 | **Cdc42ep3** | 2,29 | 0,004 |
| Tspan1 | -2,52 | 0,012 | **Erbb4** | 2,28 | 0,004 |
| Slc35f3 | -2,51 | 0,046 | **Pinc** | 2,28 | 0,007 |
| Slc10a5 | -2,50 | 0,008 | **Arhgef6** | 2,28 | 0,015 |
| Hmgcs2 | -2,49 | 0,019 | **Chst1** | 2,27 | 0,029 |
| Rasgrf1 | -2,48 | 0,021 | **Plce1** | 2,27 | 0,010 |
| Lrrc8b | -2,47 | 0,050 | **Hexb** | 2,27 | 0,007 |
| Gm13139 | -2,45 | 0,009 | **Cyp24a1** | 2,26 | 0,020 |
| Gnpnat1 | -2,41 | 0,031 | **Rnf125** | 2,25 | 0,036 |
| Man1a | -2,40 | 0,037 | **Slc28a3** | 2,25 | 0,050 |
| Hist2h3c2 | -2,39 | 0,011 | **Cebpb** | 2,24 | 0,013 |
| Gm20235 | -2,39 | 0,041 | **Igf1r** | 2,24 | 0,005 |
| Nrxn3 | -2,39 | 0,014 | **Pde7b** | 2,23 | 0,004 |
| Zfp385a | -2,39 | 0,024 | **Rbp1** | 2,23 | 0,040 |
| Cdo1 | -2,38 | 0,044 | **Ror1** | 2,22 | 0,011 |
| Lpcat4 | -2,38 | 0,018 | **Spns3** | 2,22 | 0,010 |
| Arhgap40 | -2,37 | 0,012 | **Egfr** | 2,22 | 0,005 |
| Cldn6 | -2,35 | 0,036 | **Gjb2** | 2,21 | 0,021 |
| Pls1 | -2,31 | 0,010 | **Aldh1a3** | 2,21 | 0,004 |
| 5430419D17Rik | -2,30 | 0,044 | **Hey1** | 2,20 | 0,034 |
| Trpc6 | -2,30 | 0,035 | **Kcnk5** | 2,20 | 0,007 |
| Itga5 | -2,29 | 0,020 | **Mme** | 2,19 | 0,007 |
| Fbp1 | -2,29 | 0,050 | **Crabp2** | 2,19 | 0,005 |
| Pgr | -2,28 | 0,027 | **Cck** | 2,19 | 0,004 |
| Slc16a4 | -2,28 | 0,019 | **Cytip** | 2,19 | 0,015 |
| Ccdc162 | -2,28 | 0,007 | **Wnt5b** | 2,18 | 0,005 |
| Zfp97 | -2,27 | 0,005 | **Mtss1l** | 2,18 | 0,009 |
| Snhg11 | -2,26 | 0,021 | **C2cd4b** | 2,18 | 0,005 |
| Tmem86a | -2,25 | 0,037 | **Itgb3** | 2,17 | 0,029 |
| Zbtb8a | -2,25 | 0,024 | **Ltf** | 2,17 | 0,015 |
| AW112010 | -2,24 | 0,028 | **Ltbp2** | 2,17 | 0,017 |
| Obp2a | -2,23 | 0,010 | **Chi3l1** | 2,17 | 0,010 |
| Scamp5 | -2,23 | 0,011 | **Pag1** | 2,17 | 0,004 |
| Muc16 | -2,22 | 0,045 | **Egln3** | 2,16 | 0,025 |
| Fzd4 | -2,21 | 0,012 | **Pdlim3** | 2,16 | 0,005 |
| Proc | -2,21 | 0,017 | **Fcgbp** | 2,12 | 0,005 |
| Steap1 | -2,20 | 0,010 | **Egr3** | 2,11 | 0,007 |
| Myb | -2,20 | 0,045 | **Lgr6** | 2,11 | 0,004 |
| Reg3g | -2,20 | 0,035 | **Kit** | 2,11 | 0,006 |
| 4930412O13Rik | -2,19 | 0,045 | **Tgfb2** | 2,10 | 0,007 |
| Styx | -2,17 | 0,020 | **Tfap2c** | 2,10 | 0,004 |
| Nav1 | -2,17 | 0,011 | **BC006965** | 2,09 | 0,005 |
| Hamp | -2,16 | 0,035 | **Ntn1** | 2,09 | 0,005 |
| Ptpn5 | -2,16 | 0,018 | **Snta1** | 2,08 | 0,005 |
| Tmem54 | -2,16 | 0,016 | **Mfi2** | 2,08 | 0,005 |
| Prom1 | -2,15 | 0,033 | **C3** | 2,07 | 0,010 |
| Clip4 | -2,15 | 0,008 | **Atp6v1b1** | 2,07 | 0,004 |
| Bcar3 | -2,15 | 0,019 | **Lrg1** | 2,07 | 0,008 |
| Msx2 | -2,15 | 0,023 | **Fads1** | 2,07 | 0,004 |
| Kcnma1 | -2,14 | 0,016 | **Itpkb** | 2,07 | 0,007 |
| Fer1l4 | -2,13 | 0,014 | **Gm266** | 2,06 | 0,019 |
| Cntfr | -2,13 | 0,022 | **Ogfrl1** | 2,06 | 0,007 |
| Ffar4 | -2,12 | 0,024 | **Pgm5** | 2,04 | 0,006 |
| Ccbe1 | -2,12 | 0,009 | **Ccrl2** | 2,04 | 0,020 |
| Tbc1d16 | -2,12 | 0,017 | **Lpcat1** | 2,03 | 0,025 |
| Tox2 | -2,12 | 0,009 | **Atp2b1** | 2,03 | 0,004 |
| Itih5 | -2,11 | 0,016 | **Plxdc2** | 2,03 | 0,018 |
| Jag1 | -2,09 | 0,034 | **Paqr6** | 2,03 | 0,006 |
| Dgat2 | -2,09 | 0,029 | **Map2** | 2,02 | 0,005 |
| Ralgps2 | -2,09 | 0,016 | **Pfkp** | 2,02 | 0,019 |
| Slc7a2 | -2,09 | 0,032 | **Ugt8a** | 2,02 | 0,026 |
| Eps8 | -2,08 | 0,010 | **Muc1** | 2,02 | 0,005 |
| Atp2c2 | -2,07 | 0,010 | **Pxdc1** | 2,02 | 0,007 |
| Padi2 | -2,06 | 0,033 | **Emid1** | 2,01 | 0,030 |
| Gm20016 | -2,05 | 0,014 | **Cp** | 2,01 | 0,007 |
| Snx32 | -2,04 | 0,004 | **Rasgef1c** | 2,00 | 0,006 |
| Slc25a35 | -2,04 | 0,035 | **Clic6** | 2,00 | 0,004 |
| Cd97 | -2,03 | 0,021 | **Barx2** | 2,00 | 0,013 |
| Mum1l1 | -2,03 | 0,035 | **Cyp2d22** | 2,00 | 0,030 |
| Prlr | -2,03 | 0,044 | **Muc20** | 1,99 | 0,013 |
| Ecm1 | -2,03 | 0,038 | **Ttpa** | 1,99 | 0,024 |
| Cdk18 | -2,03 | 0,019 | **Angpt1** | 1,99 | 0,004 |
| Gclc | -2,02 | 0,029 | **Car12** | 1,99 | 0,029 |
| Tmem158 | -2,02 | 0,048 | **Pim1** | 1,98 | 0,041 |
| Dzip1 | -2,02 | 0,017 | **Ptprz1** | 1,97 | 0,042 |
| Dusp10 | -2,01 | 0,027 | **Cldn1** | 1,97 | 0,031 |
| Reep6 | -2,01 | 0,036 | **Apod** | 1,96 | 0,021 |
| Stard13 | -2,01 | 0,015 | **Pdgfra** | 1,96 | 0,034 |
| Ccdc92 | -2,00 | 0,017 | **Tnfrsf11a** | 1,96 | 0,023 |
| Fgf11 | -2,00 | 0,010 | **Rapgef5** | 1,96 | 0,007 |
| Pik3c2g | -1,99 | 0,032 | **Prkg1** | 1,95 | 0,025 |
| Acot2 | -1,99 | 0,021 | **Bach2** | 1,94 | 0,015 |
| Pik3r3 | -1,97 | 0,043 | **Lama4** | 1,94 | 0,007 |
| Dscam | -1,97 | 0,025 | **Lipa** | 1,94 | 0,004 |
| F830016B08Rik | -1,97 | 0,016 | **Aqp1** | 1,94 | 0,010 |
| C2 | -1,97 | 0,020 | **Ehd3** | 1,94 | 0,004 |
| Kcnab3 | -1,97 | 0,006 | **Khdrbs3** | 1,93 | 0,008 |
| Gatsl2 | -1,97 | 0,008 | **Creb5** | 1,93 | 0,044 |
| Slc36a4 | -1,97 | 0,007 | **Olfr1158** | 1,93 | 0,034 |
| Mgat5 | -1,96 | 0,033 | **Arhgap44** | 1,93 | 0,004 |
| Slc5a6 | -1,96 | 0,017 | **Spns2** | 1,93 | 0,007 |
| Dnajc12 | -1,96 | 0,013 | **Spry2** | 1,92 | 0,005 |
| AI987944 | -1,96 | 0,005 | **Serpinh1** | 1,92 | 0,011 |
| Cbs | -1,95 | 0,017 | **Enpp1** | 1,92 | 0,007 |
| Gcnt2 | -1,95 | 0,031 | **Gm13889** | 1,92 | 0,016 |
| Slc24a3 | -1,95 | 0,005 | **Epha4** | 1,92 | 0,009 |
| Eng | -1,93 | 0,043 | **Ttc28** | 1,91 | 0,011 |
| Meis1 | -1,93 | 0,030 | **Elf5** | 1,91 | 0,015 |
| Agtr1a | -1,92 | 0,026 | **Tspan8** | 1,91 | 0,012 |
| Prkar2b | -1,92 | 0,006 | **Boc** | 1,89 | 0,011 |
| Zfp52 | -1,92 | 0,017 | **Ldhb** | 1,89 | 0,012 |
| Capsl | -1,92 | 0,037 | **Enpp3** | 1,89 | 0,005 |
| Lphn1 | -1,91 | 0,020 | **Kank4** | 1,89 | 0,010 |
| Tmem56 | -1,91 | 0,047 | **Anpep** | 1,89 | 0,007 |
| Tspan13 | -1,91 | 0,021 | **Slc16a2** | 1,88 | 0,004 |
| Tuba8 | -1,91 | 0,021 | **Rassf4** | 1,88 | 0,005 |
| Hist1h2bc | -1,91 | 0,040 | **Lrig3** | 1,88 | 0,039 |
| Phyh | -1,90 | 0,031 | **Kcnd2** | 1,88 | 0,013 |
| Rassf6 | -1,90 | 0,008 | **Vmn1r159** | 1,88 | 0,048 |
| Asic2 | -1,89 | 0,012 | **Nr4a2** | 1,87 | 0,006 |
| Sipa1l1 | -1,89 | 0,022 | **Cdhr1** | 1,87 | 0,010 |
| Stc2 | -1,88 | 0,045 | **Pik3ap1** | 1,87 | 0,030 |
| Foxred2 | -1,88 | 0,038 | **Slc43a2** | 1,87 | 0,019 |
| Slc35g2 | -1,88 | 0,043 | **Rhoj** | 1,86 | 0,022 |
| Dhcr24 | -1,87 | 0,036 | **Mgll** | 1,85 | 0,012 |
| Pllp | -1,87 | 0,007 | **Prkaa2** | 1,85 | 0,007 |
| Ano9 | -1,87 | 0,019 | **Stk10** | 1,85 | 0,010 |
| Steap2 | -1,87 | 0,027 | **Foxc1** | 1,85 | 0,005 |
| 2610524H06Rik | -1,87 | 0,024 | **P2rx7** | 1,85 | 0,005 |
| Hp | -1,87 | 0,021 | **Trf** | 1,84 | 0,021 |
| Zfp946 | -1,87 | 0,019 | **Foxi1** | 1,83 | 0,013 |
| A130040M12Rik | -1,86 | 0,030 | **Sdpr** | 1,82 | 0,013 |
| 5930412G12Rik | -1,86 | 0,019 | **March3** | 1,82 | 0,005 |
| Mvb12b | -1,86 | 0,012 | **Pitpnc1** | 1,82 | 0,009 |
| Fgfr2 | -1,86 | 0,049 | **Tmtc2** | 1,82 | 0,006 |
| Wnt4 | -1,86 | 0,030 | **Tns3** | 1,82 | 0,030 |
| Gpc1 | -1,86 | 0,014 | **Nr4a3** | 1,81 | 0,043 |
| Spred3 | -1,85 | 0,007 | **Hs3st3b1** | 1,81 | 0,005 |
| Tnxb | -1,85 | 0,018 | **Ksr1** | 1,81 | 0,010 |
| Gpr155 | -1,85 | 0,031 | **Epb4,1l4a** | 1,80 | 0,011 |
| Slc38a1 | -1,85 | 0,023 | **Ldlrad4** | 1,80 | 0,008 |
| Rab31 | -1,84 | 0,019 | **C4b** | 1,80 | 0,011 |
| Ypel4 | -1,83 | 0,010 | **Pde9a** | 1,80 | 0,006 |
| Porcn | -1,83 | 0,035 | **Ncam1** | 1,80 | 0,010 |
| sep-10 | -1,83 | 0,027 | **Gm8439** | 1,80 | 0,046 |
| Prom2 | -1,83 | 0,032 | **Cgnl1** | 1,79 | 0,028 |
| Rev3l | -1,83 | 0,022 | **Itga2** | 1,79 | 0,019 |
| Slc5a5 | -1,83 | 0,018 | **Cpe** | 1,79 | 0,010 |
| Kcnj11 | -1,82 | 0,006 | **Cd44** | 1,79 | 0,013 |
| B3gnt7 | -1,82 | 0,035 | **Slco1a5** | 1,79 | 0,007 |
| Adck5 | -1,82 | 0,008 | **Wtip** | 1,78 | 0,004 |
| Gnaz | -1,82 | 0,021 | **Tgfbr3** | 1,78 | 0,011 |
| Tbx3 | -1,82 | 0,041 | **Fam20a** | 1,78 | 0,017 |
| Zfp810 | -1,81 | 0,013 | **Plin2** | 1,76 | 0,004 |
| Fbxw17 | -1,81 | 0,018 | **AU020206** | 1,76 | 0,008 |
| Sec14l4 | -1,81 | 0,017 | **Gm2799** | 1,76 | 0,018 |
| Alox15 | -1,80 | 0,010 | **Ctnnd2** | 1,75 | 0,027 |
| Gm6712 | -1,80 | 0,007 | **Mafb** | 1,75 | 0,028 |
| Phka1 | -1,80 | 0,035 | **Itpr2** | 1,75 | 0,005 |
| Dnaaf3 | -1,80 | 0,014 | **Nynrin** | 1,74 | 0,042 |
| Hc | -1,80 | 0,007 | **Cdk6** | 1,74 | 0,006 |
| Entpd3 | -1,80 | 0,006 | **Pkp1** | 1,74 | 0,031 |
| Wfdc2 | -1,80 | 0,007 | **Dclk1** | 1,74 | 0,013 |
| Robo1 | -1,80 | 0,008 | **Rorc** | 1,74 | 0,008 |
| Lactb2 | -1,79 | 0,048 | **Papln** | 1,73 | 0,014 |
| Ube2q2 | -1,79 | 0,023 | **Atp10b** | 1,73 | 0,025 |
| Trim62 | -1,79 | 0,017 | **Ppap2b** | 1,73 | 0,035 |
| Fam69a | -1,79 | 0,029 | **Cd14** | 1,72 | 0,005 |
| Ttc9 | -1,79 | 0,035 | **Olfr577** | 1,72 | 0,018 |
| C1qtnf6 | -1,79 | 0,025 | **Rasal1** | 1,72 | 0,020 |
| Soat1 | -1,79 | 0,022 | **Olfr347** | 1,72 | 0,034 |
| Ubald2 | -1,79 | 0,007 | **Arrb1** | 1,71 | 0,005 |
| Marcks | -1,78 | 0,026 | **Stk39** | 1,71 | 0,004 |
| Dlg4 | -1,78 | 0,024 | **Ptger4** | 1,71 | 0,006 |
| Hist3h2a | -1,77 | 0,019 | **Thbs1** | 1,70 | 0,017 |
| Atp6v0e2 | -1,77 | 0,005 | **Lmo4** | 1,70 | 0,009 |
| Ror2 | -1,77 | 0,007 | **Ccdc141** | 1,70 | 0,015 |
| Iigp1 | -1,77 | 0,019 |  |  |  |
| Trp53inp2 | -1,76 | 0,034 |  |  |  |
| 4930451C15Rik | -1,76 | 0,020 |  |  |  |
| Igf2bp2 | -1,76 | 0,018 |  |  |  |
| Faah | -1,76 | 0,007 |  |  |  |
| D730039F16Rik | -1,76 | 0,007 |  |  |  |
| LOC100503186 | -1,76 | 0,026 |  |  |  |
| Lrrk1 | -1,75 | 0,025 |  |  |  |
| Esr1 | -1,75 | 0,024 |  |  |  |
| Pet112 | -1,75 | 0,010 |  |  |  |
| Adrb1 | -1,75 | 0,024 |  |  |  |
| Sptlc3 | -1,74 | 0,015 |  |  |  |
| Parp8 | -1,73 | 0,022 |  |  |  |
| Slc35f6 | -1,73 | 0,029 |  |  |  |
| Gli3 | -1,73 | 0,014 |  |  |  |
| Arg1 | -1,73 | 0,025 |  |  |  |
| Sgsm1 | -1,72 | 0,024 |  |  |  |
| Fam174b | -1,72 | 0,007 |  |  |  |
| Man2a2 | -1,72 | 0,044 |  |  |  |
| Swsap1 | -1,71 | 0,005 |  |  |  |
| Mid1 | -1,71 | 0,028 |  |  |  |
| Pde5a | -1,70 | 0,025 |  |  |  |
| Plcb1 | -1,70 | 0,018 |  |  |  |
| Gipc2 | -1,70 | 0,010 |  |  |  |
| Sox12 | -1,70 | 0,025 |  |  |  |
| Mras | -1,70 | 0,017 |  |  |  |
| Plod1 | -1,70 | 0,024 |  |  |  |
