# Supplementary material for: Luminal Progenitors Restrict Their Lineage Potential during Mammary Gland Development
Source: PLoS Biol. 2015 Feb 17;13(2):e1002069. doi: 10.1371/journal.pbio.1002069 (PMC4331521; doi:10.1371/journal.pbio.1002069)
Supplement: S2 Table — Primer sequences used for RT-PCR analysis in this work. (DOCX) [file pbio.1002069.s011.docx]

**S2 Table. Related to Experimental Procedures.**

Primer sequences used for RT-PCR analysis

| Gene Symbol | **Forward** | **Reverse** |
| --- | --- | --- |
| *18S* | GTAACCCGTTGAACCCCATT | CCATCCAATCGGTAGTAGCG |
| *Bglap3* | tctctctgctcactctgctg | agagaggacagggaggatca |
| *Btn1a1* | acgtcagagtccaagaagca | agggccaggagtcaaatctc |
| *Capn8* | cagctcaagagggtcctgaa | ccacaaagccgtcaaagtca |
| *Chst8* | tctcgtccatcctgctgttt | cggctggataaaggtttcgg |
| *Csn2* | AAAGGACTTGACAGCCATGAA | TAGCCTGGAGCACATCCTCT |
| *Cyp3a57* | acaggatccctttgtgcaga | tcaccaccatgtccaggtac |
| *GFP* | GCAAGGGCGAGGAGCTGTTCA | GTGTCGCCCTCGAACTTCAC |
| *Lalba* | ctttgcttgaatgggcctgt | tcacaacgccactgttcaag |
| *Muc13* | cgagtcagacccctaatccc | tctcttgcctgtcctcttgg |
| *Notch1* | \| TGTCAATGTTCGAGGACCAG \| \| --- \| | TGACGTCAGCATGTGAGTTG |
| *Pgr* | GCTTGCATGATCTTGTGAAACAGC | GGAAATTCCACAGCCAGTGTCC |
| *Rftn1* | tacagaccgactacatgccc | tgttgctggcactgagtttc |
| *Stac2* | cccagtttatgagacgctgc | atgatccgatctccaggctg |
| *Tnfs11* | TGTACTTTCGAGCGCAGATG | CCACAATGTGTTGCAGTTCC |
| *Wnt5a* | tctgtctttggcagggtgat | aagttcatgaggatgcgtgc |
